# Cerebral hypoperfusion reduces tau accumulation

**DOI:** 10.1002/acn3.52247

**Published:** 2024-12-02

**Authors:** Ghupurjan Gheni, Mitsuru Shinohara, Masami Masuda‐Suzukake, Akihiko Shindo, Atsushi Watanabe, Kaori Kawai, Guojun Bu, Hidekazu Tomimoto, Masato Hasegawa, Naoyuki Sato

**Affiliations:** ^1^ Department of Aging Neurobiology, Center for Development of Advanced Medicine for Dementia National Center for Geriatrics and Gerontology 7‐430 Morioka Obu Aichi 474‐8511 Japan; ^2^ Department of Neuroscience Mayo Clinic Jacksonville Florida 32224 USA; ^3^ Dementia Research Project Tokyo Metropolitan Institute of Medical Science 2‐1‐6 Kamikitazawa, Setagaya‐ku Tokyo 156‐8506 Japan; ^4^ Department of Neurology, Graduate School of Medicine Mie University 1577 Kurima Machiyacho Tsu City Mie 514‐8507 Japan; ^5^ Equipment Management Division, Center for Core Facility Administration, National Center for Geriatrics and Gerontology 7‐430 Morioka Obu Aichi 474‐8511 Japan; ^6^ Division of Life Science The Hong Kong University of Science and Technology Hong Kong China

## Abstract

**Objective:**

Alzheimer's disease (AD) often coexists with cerebrovascular diseases. However, the impact of cerebrovascular diseases such as stroke on AD pathology remains poorly understood.

**Methods:**

This study examines the correlation between cerebrovascular diseases and AD pathology. The research was carried out using clinical and neuropathological data collected from the National Alzheimer's Coordinating Center (NACC) database and an animal model in which bilateral common carotid artery stenosis surgery was performed, following the injection of tau seeds into the brains of wild‐type mice.

**Results:**

Analysis of the NACC database suggests that clinical stroke history and lacunar infarcts are associated with lower neurofibrillary tangle pathology. An animal model demonstrates that chronic cerebral hypoperfusion reduces tau pathology, which was observed in not only neurons but also astrocytes, microglia, and oligodendrocytes. Furthermore, we found that astrocytes and microglia were activated in response to tau pathology and chronic cerebral hypoperfusion. Additionally, cerebral hypoperfusion increased a lysosomal enzyme, cathepsin D.

**Interpretation:**

These data together indicate that cerebral hypoperfusion reduces tau accumulation likely through an increase in microglial phagocytic activity towards tau and an elevation in degradation through cathepsin D. This study contributes to understanding the relationship between tau pathology and cerebrovascular diseases in older people with multimorbidity.

## Introduction

Dementia is the loss of cognitive functioning that interferes with a person's daily life and activities. It has become a significant problem in today's aging society. There are several different forms of dementia, including Alzheimer's disease (AD), Lewy body dementia (LBD), vascular dementia (VaD), frontotemporal lobar dementia (FTD), argyrophilic grain dementia (AGD), corticobasal degeneration (CBD), and progressive supranuclear palsy (PSP). Among them, AD, FTD, AGD, CBD, and PSP are different forms of tauopathies due to the accumulation of aggregated tau protein in neurons and glial cells in the brain.^1^, ^2^ Tau is a microtubule‐associated protein expressed predominantly in the axons of neurons.^3^, ^4^ Soluble tau is natively unfolded, whereas assembled tau forms amyloid filaments with a β‐sheet structure.^5^, ^6^

In the aging society, multiple health conditions, known as multimorbidity, are also expected among older individuals. Approximately 5–12% of older people experience a stroke,^7^, ^8^ and AD and stroke are two conditions that frequently coexist.^9^ Stroke is characterized as a neurological disorder attributed to an acute focal injury of the central nervous system by a vascular cause including cerebral infarction, intracerebral hemorrhage, and subarachnoid hemorrhage.^10^ Autopsy findings in AD patients reveal cerebral infarction in 8–35% of cases.^11^, ^12^ There is an intricate interconnection between stroke and chronic cerebral hypoperfusion. Recent studies underscore the role of chronic cerebral hypoperfusion in neurodegeneration and dementia, particularly in AD.^13^, ^14^ Indeed, cerebral hypoperfusion is frequently observed in AD.^15^, ^16^, ^17^ However, the exact impact of stroke and chronic cerebral hypoperfusion on tau pathology remains poorly understood, raising a fundamental question about how cerebrovascular diseases influence the accumulation of abnormal proteins, such as tau.

Recently, a model in which aggregated tau is injected into the brains of tau‐overexpressing mice has been developed, successfully replicating tau pathology such as neurofibrillary tangles (NFT).^18^, ^19^, ^20^ Furthermore, even in wild‐type mice, tau pathology can be reproduced by injecting NFTs from AD brains^21^, ^22^ or recombinant tau treated with dextran sulfate.^23^ On the other hand, as a model for cerebral hypoperfusion, the bilateral carotid artery stenosis (BCAS) model has been developed using micro‐coil placement on bilateral common carotid arteries.^24^, ^25^

This study first aims to clarify the correlation between cerebral vascular disease and tau accumulation by analyzing the NACC database. Then, we conducted BCAS surgery following the injection of tau seeds into the brains of wild‐type mice to examine whether chronic cerebral hypoperfusion affects the accumulation and propagation of tau pathology.

## Materials and Methods

### Clinical data analysis

#### The NACC dataset

The clinical and neuropathological data in the NACC database, which were collected by the 34 past and present Alzheimer's Disease Centers (ADCs) from September 2005 to September 2018 as the longitudinal Uniform Data Set^26^ and NACC neuropathology data‐collection form,^27^ were assessed in this study. NACC subjects are regarded as a referral‐based or volunteer case series, and the majority of the subjects are Caucasians and then Black or African Americans, consisting of non‐demented and demented subjects. There were 4744 subjects whose neuropathology data were available. As we aim to address the effects of clinical stroke or vascular pathologies on AD‐related pathologies in the relatively elderly population, we removed subjects whose age at death was below 60 years old (*n* = 220, 4.6% of whole subjects), resulting in 4524 subjects for the analysis. To assess the remote clinical history of stroke, we analyzed the variable “CBSTROKE” in the Uniform Data Set. We excluded 12 subjects whose CBSTROKE values were unavailable at all visits (0.26% of whole subjects), resulting in 4512 subjects. If a subject showed CBSTROKE = 0 (Absent) at all visits, we classified such a person into the “non‐stroke” group (*n* = 4008). If a subject showed CBSTROKE = 2 (Remote/Inactive) at any visit, we classified such a person into the “remote stroke” group (*n* = 400). In addition, when a subject showed CBSTROKE = 1 (Recent/Active) at their last visit and more than 3 years had passed by the time of neuropathological assessment, we classified the individual into the “remote stroke” group (*n* = 27). Their demographics and pathological characteristics are shown in Table 1. Neuropathology scores were evaluated as we did previously.^28^, ^29^ The information on diffuse plaques, neuritic plaques, and cerebral amyloid angiopathy (CAA) was extracted from the variables “NACCDIFF,” “NACCNEUR,” and “NACCAMY,” respectively, those were each classified into four stages, and NFT pathology was extracted from the variable “NACCBRAA” that was classified into seven stages, according to the generally‐used criteria.^30^, ^31^, ^32^ Regarding vascular pathologies, we evaluated following variables, “NACCARTE” (Arteriolosclerosis; 0 = none, 1 = mild, 2 = moderate, 3 = severe), “NACCVAS” (Atherosclerosis of the circle of Willis; 0 = none, 1 = mild, 2 = moderate, 3 = severe), “NACCVASC” (Ischemic, hemorrhagic, or vascular pathology present; 0 = no, 1 = yes), “NPLINF” (Large arterial infarcts present; 0 = no, 1 = yes), “NACCINF” (Infarcts and lacunes; 0 = no, 1 = yes), “NPLAC” (One or more lacunes; 0 = no, 1 = yes), “NPHEM” (Single or multiple hemorrhages present; 0 = no, 1 = yes), “NPMICRO” (Multiple microinfarcts present; 0 = no, 1 = yes), “NACCMICR” (Microinfarcts; 0 = no, 1 = yes), “NPWMR” (White matter rarefaction; 0 = none, 1 = mild, 2 = moderate, 3 = severe), and “NPART” (Subcortical arteriosclerotic leukoencephalopathy present; 0 = no, 1 = yes), after removing subjects whose vascular pathology scores were missing or unavailable. To define the cognitive/dementia status, and Alzheimer's type dementia status, we used “NACCUDSD” and “NACCALZD” variables, respectively. The former variable describes the cognitive/dementia status (cognitive normal, dementia, mild cognitive impairment (MCI), or impaired but not MCI (other)), and the latter variable describes the etiologic diagnosis of AD. APOE4 genotype status were defined as APOE4 carrier (ε3/ε4 or ε4/ε4) or non‐APOE4 carrier (ε2/ε2, ε2/ε3, or ε3/ε3). The statuses of hypertension, hypercholesterolemia, and diabetes were defined as we described previously.^28^, ^29^

**Table 1:** Demographic and pathological characteristics of subjects with or without remote stroke history in the NACC database.

	Total (includes recent stroke history)	Non‐stroke	Remote stroke history	*P*‐value
Number	4512	4008	427	
Age (y)	82.5 ± 8.9	81.3 ± 10.1	85.4 ± 8.3	<0.001
Male: Female	2420: 2092	2151: 1857	230: 197	0.938
RACE (While: Black: others)	4281: 163: 68	3820: 129: 59	390: 30: 7	0.001
APOE4+	1895 (43.2%)	1712 (44.0%)	160 (38.3%)	0.025
Dementia: Non‐Dementia	3500: 1012	3111: 897	335: 92	0.693
AD‐type dementia	2630	2323 (58.0%)	265 (62.1%)	0.101
CERAD diffuse plaque (none: sparse: moderate: frequent)	581: 566: 713: 2270	511: 474: 627: 2055	57: 77: 72: 181	<0.001
CERAD neuritic plaque (none: sparse: moderate: frequent)	948: 613: 905: 2047	832: 525: 789: 1853	96: 71: 97: 162	0.008
Braak NFT (stage 0 to 6)	212: 353: 500: 462: 627: 843: 1460	197: 314: 440: 386: 528: 740: 1344	13: 32: 53: 56: 81: 86: 99	<0.001
Cerebral amyloid angiopathy (none: mild: moderate: severe)	1743: 1293: 896: 490	1523: 1170: 802: 427	177: 107: 79: 52	0.188
Arteriolosclerosis (none: mild: moderate: severe)	762: 1416: 1232: 559	699: 1281: 1071: 461	47: 114: 140: 81	<0.001
Atherosclerosis of the circle of Willis (none: mild: moderate: severe)	909: 1716: 1288: 556	842: 1554: 1105: 457	50: 133: 152: 88	<0.001
Ischemic, hemorrhagic, or vascular pathology present (no: yes)	85: 4383	83: 3874	1: 422	0.001
Large arterial infarcts present (no: yes)	2390: 279	2150: 184	190: 76	<0.001
Infarcts and lacunes present (no: yes)	3585: 923	3324: 671	217: 208	<0.001
One or more lacunes present (no: yes)	2182: 487	1974: 359	166: 101	<0.001
Single or multiple hemorrhages present (no: yes)	2505: 165	2505: 165	243: 24	0.026
Multiple microinfarcts present (no: yes)	2169: 502	1940: 396	186: 80	<0.001
Microinfarcts present (no: yes)	3562: 954	3230: 773	278: 147	<0.001
White matter rarefaction (none: mild: moderate: severe)	676: 469: 300: 160	623: 420: 264: 139	48: 43: 31: 19	0.145
Subcortical arteriosclerotic leukoencephalopathy present (no: yes)	2251: 408	1979: 345	219: 47	0.232

Data are shown as the distribution or frequency, except for that of age, which are shown as the mean ± SD. *P*‐values are from the chi‐square test, except for that of age, which are from one‐way ANOVA.

#### Statistical analysis

Effects of clinical stroke history on neuropathological scores were analyzed using a multivariable linear regression model including the following co‐variants; sex, race, age at death, APOE4 status, cognitive/dementia status, and/or cardiovascular factors, including hypertension, hypercholesterolemia, and diabetes. We also analyzed the effects of stroke within AD‐type dementia by multivariable linear regression models using these co‐variants. We obtained estimates with 95% confidence intervals and associated *P*‐values, which are reported in each Table. To strictly evaluate the effects of clinical strokes on AD‐related pathologies, we multiplied raw *P*‐values by four (the number of items in our primary evaluation (diffuse plaques, neuritic plaques, Braak NFT, and CAA)) to calculate corrected p‐values with Bonferroni adjustment. To strictly evaluate vascular pathologies as the secondary evaluation, we multiplied raw p‐values by 11 (number of items of vascular pathology) to calculate corrected p‐values with Bonferroni adjustment. Our conclusions were drawn from the data that was significant even after such Bonferroni adjustments. *P*‐values less than 0.05 were significant. All statistical analyses were performed using JMP Pro software (version 14.3, SAS Institute Inc. Cary, USA).

### Animal study

#### Animals

The mice were group housed without enrichment structures in a specific pathogen‐free environment in ventilated cages and were used in the experiments according to the Guideline for the Care and Use of Laboratory Animals of our research facilities.

#### Preparation of recombinant tau protein and tau seeds

Full‐length murine 1N4R tau was prepared as previously described.^23^, ^33^, ^34^ Briefly, tau proteins were expressed in Escherichia coli BL21 cells. Following centrifugation of the cell suspension, pellets were lysed in a buffer (50 mM PIPES, pH 6.9, 1 mM EGTA, 1 mM dithiothreitol (DTT), and 0.5 mM phenylmethylsulfonyl fluoride) and sonicated on ice. The lysates were centrifuged (21,000 g, 15 min, at 4°C) and the supernatants were boiled in the presence of 1% 2‐mercaptoethanol for 5 min. Heat‐stable fractions were applied to an SP‐Sepharose ion‐exchange chromatography column (GE Healthcare), and tau protein was eluted with 0.35 M NaCl in the same buffer. After precipitation with 50% saturated ammonium sulfate, tau protein was dialyzed against 30 mM Tris–HCl, pH 7.5. Following ultracentrifugation (113,000 *g*, 20 min.), the supernatant was utilized as soluble monomeric tau. Protein concentration was determined based on absorbance at 215 nm using reverse‐phase high‐pressure liquid chromatography with an Aquapore RP300 column (PerkinElmer) and double‐checked using a NanoDrop 2000 spectrophotometer (Thermo Fisher Scientific). Tau seeds were prepared as reported previously.^23^ Soluble tau protein (4.5 mg/mL) was incubated in the presence of 200 mg/mL dextran sulfate (Sigma‐Aldirich), 5 mM dithiothreitol, 0.1% NaN_3_ in 30 mM Tris–HCl, pH 7.5 with shaking at 200 rpm at 37°C for 7 days. Tau fibrils were collected by ultracentrifuge at 113,000 *g* for 20 min, washed with saline, and spun again. Tau seeds were resuspended with saline, fragmented by sonication using a Sonifier SFX250 cup horn sonicator (BRANSON), and stored at −80°C until use.

#### Stereotactic tau injection

Three‐month‐old male C57BL/6J (Japan SLC) mice were used for the experiment. Animals were group housed (four or five animals per cage) with free access to food and water. The tau propagation model by intracerebral injection of dextran sulfate (DS)‐induced tau seeds was described previously.^23^ Anesthesia was performed using intraperitoneal injection with a combination of three anesthetics: 0.3 mg/kg medetomidine hydrochloride (Nippon Zenyaku Kogyo, Fukushima, Japan), 4.0 mg/kg midazolam (Astellas, Tokyo, Japan), and 5.0 mg/kg butorphanol tartrate (Meiji Seika Pharma, Gifu, Japan), and then 5 μl (90 μM) dextran sulfate‐induced tau seeds (5 μl PBS for control) were injected unilaterally into the hippocampus (anterior–posterior: −2.5 mm; medial‐lateral: 2 mm; dorsal‐ventral: −2.3 mm from the bregma and dura). After the injection of tau seeds, 3.0 mg/kg atipamezole hydrochloride (Nippon Zenyaku Kogyo), an antagonist of medetomidine hydrochloride, was injected intraperitoneally to promote safe awakening. To maintain body temperature during surgery, the mice were warmed with a heating pad at 37°C on a stereotaxic frame.

#### BCAS surgery

Surgical procedures for BCAS were described previously.^24^, ^35^ After 2 months of tau injection, mice were anesthetized (as described above) and then underwent BCAS to induce chronic cerebral hypoperfusion, or sham operation, in which the common carotid artery was exposed and gently freed from its sheath. For BCAS‐treated mice, a micro‐coil with a 0.18‐mm inner diameter (manufactured by Samini, Shizuoka, Japan) was attached to the common carotid artery bilaterally. After the surgery, the mice were awakened using the same method described above. To maintain the body temperature of the mice after surgery, they were warmed with a heating pad set to 37°C.

#### Behavioral experiments

Behavioral experiments were conducted to assess the locomotion activity, spatial learning, and memory abilities of the mice, as we described previously with some modifications.^35^ The locomotion activity was measured using the open‐field test, in which mice were placed in a cubical plastic box (30 × 30 × 30 cm^3^, width × length × height) and allowed to roam freely for 15 min. Spatial learning and memory abilities were evaluated using Morris water maze tests. The test was conducted in a circular pool (120 cm diameter) filled with water adjusted to ambient temperature. For hidden platform training, a transparent platform (10 cm diameter) was submerged 1.5 cm below the water level. The pool was in a test room with many external cues. From day 1 to day 4, four training trials (two sessions) were performed each day. During each trial, the mice were released from four pseudo‐randomly assigned starting points and allowed to swim for 60 sec. The inter‐trial interval was approximately 10 min, and the intersession interval was 2 h. After mounting the platform, the mice were allowed to remain there for 10 sec and were then placed in a holding cage with a heating lamp until the start of the next trial. If a mouse failed to find the platform, it was guided to the platform and allowed to rest on the platform for 10 sec. The probe test was performed 24 h after the last hidden platform training. In the probe test, the platform was removed, and the mouse was released from the opposite quadrant and allowed to swim freely for 60 sec. In the visible platform test performed after the last probe test on the same day, the platform was elevated above the water surface. The mice underwent three trials lasting 60 sec. All experiments were conducted at approximately the same time each day. An overhead camera was used to track the movement of mice in both the open‐field and Morris water maze tests, and the video was analyzed using the ANY‐maze software (Muromachi Kikai, Tokyo, Japan).

#### Tissue collection and preparation

Four more months after BCAS surgery, mice were anesthetized with isoflurane (Fujifilm Wako Pure Chemicals, Osaka, Japan) and perfused transcardially with phosphate‐buffered saline (PBS) plus complete protease inhibitor cocktail (Roche Diagnostics, Basel, Switzerland). After perfusion, the brain was removed from the skull and was fixed with 4% paraformaldehyde/PBS overnight and sectioned at 50‐μm thickness using a VT1200 vibratome (Leica, Heidelberg, Germany) for histological analysis. All experiments were carried out in agreement with the Guidelines for Proper Conduct of Animal Experiments (Science Council of Japan) and the ARRIVE guidelines, and all experimental protocols were approved by the National Center for Geriatrics and Gerontology, Aichi, Japan.

#### Immunohistochemistry and immunofluorescence

Free‐floating sections were mounted on glass slides and processed for antigen retrieval by heating at microwave, 500 w, in 0.1 M sodium citrate buffer, pH 6.0, for 10 min and by immersing in 95% formic acid for 10 minutes.^23^ Sections were then treated with 3% hydrogen peroxide in methanol to inactivate endogenous peroxidases, permeabilized with 0.5% Triton X‐100 in phosphate‐buffered saline (PBS), and blocked with 10% fetal bovine serum (FBS) in PBS with 0.3% Triton X‐100 (blocking buffer). Primary antibodies such as anti‐phospho‐tau (pS202/pT205) (AT8) mouse monoclonal antibody, anti‐Glial fibrillary acidic protein (GFAP) rabbit polyclonal antibody, anti‐Ionized Calcium Binding Adaptor Molecule 1 (Iba1) rabbit polyclonal antibody, and Anti‐cathepsin D (CTSD) rabbit monoclonal antibody (Table S4) in blocking buffer were incubated with sections at 4°C, overnight. For immunohistochemistry, secondary antibodies (HRP‐conjugated anti‐Mouse or anti‐Rabbit IgG) were added and incubated for 2 h at room temperature. After three washes in PBS, HRP‐labeled slides were treated with Impact DAB (Vector Laboratories), a peroxidase substrate. Following counterstaining with hematoxylin, the sections were coverslipped. For quantitative analyses, images were taken with a BZ‐X710 microscope (Keyence, Osaka, Japan), and positive areas in the selected region were measured by BZ‐X analyzer software.

For immunofluorescence, primary antibody‐bound sections were incubated at room temperature with anti‐rabbit IgG Alexa Fluor 488 or anti‐mouse IgG Alexa Fluor 546. Sections were mounted in Entellan New (Merck Millipore) or the VECTASHIELD Vibrance Antifade Mounting Medium (#H‐1700‐10; Vector Laboratories) and images were acquired using either an LSM700 or an LSM780 confocal laser‐scanning microscope (Carl Zeiss, Germany) and analyzed under a Leica TCS SP8 confocal microscope (Leica Microsystems CMS GmbH, Mannheim, Germany). Laser and detection settings were kept consistent during acquiring immunofluorescence images. Quantitative analyses were performed by ImageJ Fiji software using Z‐stack confocal images which were reconstructed with a maximum intensity projection.

#### Klüver‐Barrera staining

Sections were immersed in Luxol Fast Blue solution (Muto Chemical, Tokyo, Japan) at 65°C overnight.^35^, ^36^ Sections were chilled at room temperature, rinsed in 95% ethanol, treated with 0.1% lithium carbonate, rinsed in 70% ethanol, counterstained with 0.1% Cresyl Violet Acetate (Muto Chemical) containing 10% acetic acid, and mounted with Entellan New (Merck Millipore). Sections were analyzed on a BZ‐X810 microscope.

Using images of the white matter area (corpus callosum), the severity of white matter lesions was classified into four grades as described in Wakita's study^37^: normal (grade 0), disarrangement of nerve fibers (grade 1), formation of marked vacuoles (grade 2), and disappearance of myelinated fibers (grade 3). Three individuals performed grading in a blinded manner.

#### Statistical analyses

All statistical analyses were performed using the JMP Pro software (version 14.3, SAS Institute). All experiments were analyzed through one‐way ANOVA followed by the Tukey Kramer test/Steel‐Dwass test or repeated‐measures one‐way ANOVA followed by the Tukey HSD test.

## Results

### Clinical history of remote stroke and lacune pathology are associated with fewer Alzheimer's pathologies

**Figure 1:**
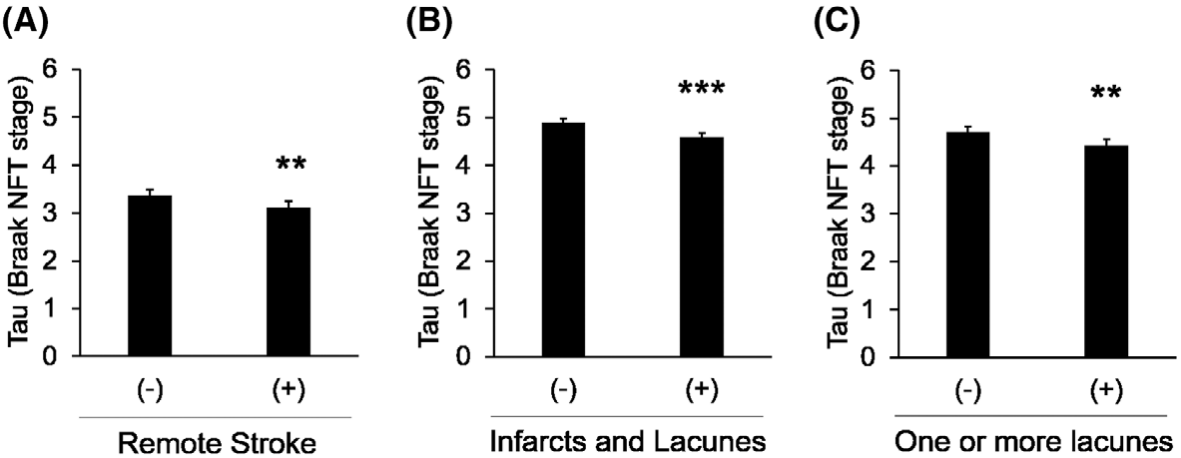
Clinical stroke and lacune pathology are associated with fewer tau pathologies. (A) Effects of remote stroke history on Braak NFT stage in whole neuropathologically‐examined subjects (*n* = 4512), adjusted for age at death, sex, race, APOE4, and cognitive/dementia status. Effects of vascular pathologies, “infarcts and lacunes” (B) or “One or more lacunes” (C), on Braak NFT stage in subjects with AD‐type dementia (*n* = 2630), adjusted for age at death, sex, race, and APOE4. Data are presented as the adjusted mean ± standard error of the mean. **P < 0.01, and ***P < 0.001; by Student's *t*‐test.

To assess the relationship between AD and cerebrovascular diseases at the clinical level, we analyzed the NACC dataset, which contains more than 4000 subjects with both clinical and neuropathological datasets. By using these datasets, we recently succeeded in analyzing the effects of APOE genotype, diabetes, and obesity on AD and vascular‐related pathological features.^28^, ^29^ Among older subjects whose neuropathological data were available, there were 427 subjects with a remote clinical history of stroke and 4008 subjects who were absent from stroke in the clinical record (see method). Of note, subjects with remote stroke history showed an increase in their age at death (Non‐stroke group; 81.3 ± 10.1 years old vs. Remote stroke group; 85.4 ± 8.3 years old, *P* < 0.001). Also, while there were no significant differences in sex and dementia status between them, the frequency of APOE4 status was relatively decreased in the remote stroke group (Non‐stroke group; 44.0% vs. Remote stroke group; 38.3%, *P* = 0.025). Controversies exist regarding whether APOE is a risk for stroke, in which the effect might be different depending on the stroke subtype or the presence of dementia.^38^, ^39^, ^40^, ^41^ At least, the current data indicates the need to adjust for APOE4 status, in addition to other general co‐variants. Also, there were several differences in pathological characteristics between non‐stroke and remote stroke groups, which might be affected by these co‐variants (Table 1). However, unexpectedly, in the model that includes age, sex, race, and APOE4, as co‐variants, we found that remote stroke history was associated with less diffuse plaques (Estimate = −0.16, 95% CI = −0.27 to −0.04, *P* = 0.0071). Similarly, remote stroke history tended to be associated with fewer neuritic plaques, and fewer Braak NFT stages, while not significant (Table 2: model 1). As the cognitive or dementia might affect these pathologies, we next included cognitive/dementia status as an additional co‐variant. In this second model, remote stroke history was still associated with less diffuse plaques (Estimate = −0.16, 95% CI = −0.27 to −0.05, *P* = 0.0032). Further, this stroke history was significantly associated with fewer neuritic plaques (Estimate = −0.20, 95% CI = −0.31 to −0.09, *P* = 0.0005), and Braak NFT stages (Estimate = −0.26, 95% CI = −0.43 to −0.09, *P* = 0.003) (Table 2: model 2). The effect of remote stoke history on NFT was also visualized as a graph (Fig. 1A). To exclude the possibility that the existence of vascular‐type dementia, which would have fewer Alzheimer's pathologies, might affect our results, we then focused on subjects who were diagnosed with AD‐type dementia. In this third model, remote stroke history was still associated with less diffuse plaques (Estimate = −0.20, 95% CI = −0.32 to −0.08, *P* = 0.0011), neuritic plaques (Estimate = −0.22, 95% CI = −0.34 to −0.11, *P* = 0.0002), and Braak NFT stages (Estimate = −0.28, 95% CI = −0.46 to −0.10, *P* = 0.0027) (Table 2: model 3). We also adjusted for the effects of cardiovascular factors, hypertension, hypercholesterolemia, and diabetes statuses, and observed similar effects of remote stroke history (Table 2: model 4). On the other hand, as expected, remote stroke history was generally associated with more vascular pathologies, including arteriolosclerosis, infarcts, hemorrhages, and lacunes (Table 2).

**Table 2:** Effects of remote stroke history on Alzheimer's and vascular pathologies in several models.

Effects of remote stroke history	Model 1		Model 2		Model 3		Model 4	
	Estimate^a^	*P*‐value	Estimate^a^	*P*‐value	Estimate^a^	*P*‐value	Estimate^a^	*P*‐value
On Alzheimer's pathologies								
CERAD diffuse plaque (0 = none, 1 = sparse, 2 = moderate, 3 = frequent)	−0.16 (−0.27, −0.04)	0.0071	−0.16 (−0.27, −0.05)	0.0032	−0.2 (−0.32, −0.08)	0.0011	−0.22 (−0.36, −0.07)	0.0042
CERAD neuritic plaque (0 = none, 1 = sparse, 2 = moderate, 3 = frequent)	−0.1 (−0.21, 0.02)	0.0928	−0.2 (−0.31, −0.09)	0.0005	−0.22 (−0.34, −0.11)	0.0002	−0.23 (−0.38, −0.09)	0.0014
Braak NFT (stage 0 to 6)	−0.14 (−0.33, 0.04)	0.1214	−0.26 (−0.43, −0.09)	0.003	−0.28 (−0.46, −0.1)	0.0027	−0.26 (−0.48, −0.04)	0.0185
Cerebral amyloid angiopathy (CAA) (0 = none,1 = mild, 2 = moderate, 3 = severe)	0 (−0.1, 0.1)	0.9603	−0.02 (−0.12, 0.08)	0.6428	−0.03 (−0.16, 0.1)	0.6613	−0.1 (−0.25, 0.05)	0.2096
On Vascular pathologies								
Arteriolosclerosis (0 = none,1 = mild, 2 = moderate, 3 = severe)	0.26 (0.15, 0.36)	<0.0001	0.23 (0.13, 0.33)	<0.0001	0.19 (0.06, 0.32)	0.0046	0.2 (0.04, 0.36)	0.0119
Atherosclerosis of the circle of Willis (0 = none,1 = mild, 2 = moderate, 3 = severe)	0.24 (0.15, 0.33)	<0.0001	0.23 (0.14, 0.32)	<0.0001	0.27 (0.15, 0.39)	<0.0001	0.22 (0.09, 0.36)	0.0013
Ischemic, hemorrhagic, or vascular pathology present (0 = no, 1 = one or more vascular pathology)	0.02 (0, 0.03)	0.0363	0.01 (0, 0.03)	0.0603	0.01 (0, 0.03)	0.1103	0.01 (−0.01, 0.03)	0.2663
Large arterial infarcts present (0 = no, 1 = yes)	0.19 (0.16, 0.23)	<0.0001	0.19 (0.16, 0.23)	<0.0001	0.19 (0.14, 0.24)	<0.0001	0.21 (0.16, 0.26)	<0.0001
Infarcts and lacunes (0 = no, 1 = yes)	0.3 (0.26, 0.33)	<0.0001	0.29 (0.25, 0.33)	<0.0001	0.29 (0.24, 0.34)	<0.0001	0.33 (0.27, 0.39)	<0.0001
One or more lacunes (0 = no, 1 = yes)	0.2 (0.16, 0.25)	<0.0001	0.2 (0.15, 0.25)	<0.0001	0.22 (0.16, 0.28)	<0.0001	0.21 (0.14, 0.28)	<0.0001
Single or multiple hemorrhages present (0 = no, 1 = yes)	0.03 (0, 0.06)	0.0277	0.04 (0.01, 0.07)	0.0208	0.04 (0, 0.08)	0.0447	0.04 (−0.01, 0.08)	0.0891
Multiple microinfarcts present (0 = no, 1 = yes)	0.11 (0.07, 0.16)	<0.0001	0.12 (0.07, 0.17)	<0.0001	0.07 (0.01, 0.13)	0.0286	0.07 (0, 0.14)	0.0469
Microinfarcts (0 = no, 1 = yes)	0.12 (0.08, 0.16)	<0.0001	0.12 (0.08, 0.16)	<0.0001	0.08 (0.03, 0.13)	0.002	0.11 (0.04, 0.17)	0.0008
White matter rarefaction (0 = none,1 = mild, 2 = moderate, 3 = severe)	0.23 (0.05, 0.4)	0.0123	0.2 (0.03, 0.38)	0.0234	0.07 (−0.16, 0.3)	0.5505	0 (−0.32, 0.33)	0.9865
Subcortical arteriosclerotic leukoencephalopathy present (0 = no, 1 = yes)	0.02 (−0.02, 0.07)	0.3299	0.02 (−0.03, 0.07)	0.3851	0.03 (−0.03, 0.1)	0.2719	0.04 (−0.03, 0.11)	0.2383

Model 1 includes sex, age, race, and APOE4 as co‐variants. Model 2 includes sex, age, race, APOE4, and cognitive/dementia status as co‐variants. Model 3 includes sex, age, race, and APOE4 as co‐variants within AD‐type dementia. Model 4 includes sex, age, race, APOE4, hypertension, hypercholesterolemia, and diabetes statuses as co‐variants within AD‐type dementia. The blue color indicates the negative effects with statistical significance, while the red color indicates the positive effects with statistical significance.

^a^ Estimate with 95% confidence interval.

To further study such inverse associations between AD and cerebrovascular diseases, we then assessed the relationship between individual vascular pathologies and AD pathologies. In the model adjusting for age, sex, race, APOE4, and cognitive/dementia status, we observed different trends of association depending on each vascular pathology; “arteriolosclerosis,” “atherosclerosis of the circle of Willis,” and “presence of any vascular pathology” were associated with more AD pathologies, including diffuse plaques, neuritic plaques, and NFT, while “large arterial infarcts,” “infarcts and lacunes,” and “one or more lacunes” tended to be associated with less these AD‐related pathologies (Table S1). Such trends were more remarkable and statistically significant in the second model, which was limited to only subjects with AD‐type dementia (Table 3). The effect of “infarcts and lacunes” and “one or more lacunes” on NFT in subjects with AD‐type dementia were visualized as a graph (Fig. 1B and C). Effects of other vascular pathologies, including hemorrhages, microinfarcts, and white matter rarefaction are not fully apparent, while subcortical arteriosclerotic leukoencephalopathy was associated with less diffuse and neuritic plaques. Notably, on the other hand, most vascular pathologies were associated with more CAA (Table 3 and Table S1). These results of the impacts of cerebrovascular diseases on CAA might be consistent with a previous study analyzing autopsy brains and animal models.^42^

**Table 3:** Effects of vascular pathologies on Alzheimer's pathologies in the model include sex, age, race, and APOE4 as co‐variants within AD‐type dementia.

Effects of vascular pathologies	On diffuse plaques		On neuritic plaques		On Braak NFT		On CAA	
	Estimate^a^	*P*‐value	Estimate^a^	*P*‐value	Estimate^a^	*P*‐value	Estimate^a^	*P*‐value
Arteriolosclerosis (0 = none,1 = mild, 2 = moderate, 3 = severe)	0.04 (0, 0.08)	0.0602	0.05 (0.01, 0.09)	0.0131	0.09 (0.02, 0.15)	0.0066	0.21 (0.17, 0.25)	<0.0001
Atherosclerosis of the circle of Willis (0 = none,1 = mild, 2 = moderate, 3 = severe)	0.07 (0.03, 0.11)	0.001	0.09 (0.05, 0.13)	<0.0001	0.1 (0.04, 0.16)	0.0013	0.06 (0.02, 0.11)	0.0051
Ischemic, hemorrhagic, or vascular pathology present (0 = no, 1 = one or more vascular pathology)	0.49 (0.15, 0.83)	0.0044	0.57 (0.24, 0.9)	0.0008	1.4 (0.9, 1.91)	<0.0001	1.15 (0.79, 1.51)	<0.0001
Large arterial infarcts present (0 = no, 1 = yes)	−0.09 (−0.26, 0.08)	0.2876	−0.24 (−0.4, −0.08)	0.0031	−0.2 (−0.44, 0.05)	0.1131	0.2 (0.03, 0.38)	0.0207
Infarcts and lacunes (0 = no, 1 = yes)	−0.11 (−0.2, −0.02)	0.0174	−0.15 (−0.24, −0.06)	0.0007	−0.31 (−0.45, −0.18)	<0.0001	0.12 (0.02, 0.22)	0.018
One or more lacunes (0 = no, 1 = yes)	−0.15 (−0.27, −0.02)	0.0242	−0.13 (−0.25, −0.01)	0.0356	−0.29 (−0.48, −0.1)	0.003	0.02 (−0.11, 0.16)	0.7153
Single or multiple hemorrhages present (0 = no, 1 = yes)	0.04 (−0.17, 0.25)	0.7156	0.06 (−0.14, 0.26)	0.5632	−0.09 (−0.4, 0.21)	0.5467	0.25 (0.04, 0.46)	0.0214
Multiple microinfarcts present (0 = no, 1 = yes)	0.04 (−0.09, 0.16)	0.5842	−0.09 (−0.21, 0.04)	0.171	−0.08 (−0.27, 0.11)	0.4078	0.15 (0.02, 0.29)	0.0213
Microinfarcts (0 = no, 1 = yes)	0.04 (−0.05, 0.12)	0.4133	−0.08 (−0.17, 0.01)	0.0732	−0.09 (−0.23, 0.04)	0.17	0.13 (0.03, 0.22)	0.0094
White matter rarefaction (0 = none,1 = mild, 2 = moderate, 3 = severe)	0.04 (−0.01, 0.09)	0.1217	0.06 (0, 0.11)	0.0351	0.06 (−0.02, 0.14)	0.1531	0.16 (0.09, 0.22)	<0.0001
Subcortical arteriosclerotic leukoencephalopathy present (0 = no, 1 = yes)	−0.26 (−0.39, −0.12)	0.0002	−0.29 (−0.42, −0.16)	<0.0001	−0.03 (−0.23, 0.17)	0.7833	−0.03 (−0.17, 0.11)	0.6338

The blue color indicates the negative effects with statistical significance, while the red color indicates the positive effects with statistical significance.

^a^ Estimate with 95% confidence interval.

As one of the secondary analyses, we also evaluated a series of pathological variables that recorded the number and size of old infarcts observed grossly in each brain area (cerebral cortex, subcortical/periventricular white matter, deep cerebral gray matter or internal capsule, and brainstem or cerebellum). While there were many subjects whose information was missing, the remote stroke history group showed more number and size of old infarcts in each brain area (Table S2). The number and size of old infarcts in each brain area generally showed similar trends of associations with AD‐related pathology, while there were some differences depending on areas and amyloid/NFT (Table S3). As the number of subjects with available data is relatively small, it would be difficult to draw a conclusion from this analysis to assess the effects of the number and size of infarcts on AD‐related pathology in each area.

Collectively, in the NACC dataset, we observed inverse associations of AD‐related pathologies (senile plaques and tau) with remote stroke history, and some specific vascular pathologies, including infarcts and lacunes, potentially in widespread brain areas. This association was still observed after adjusting for cognitive/dementia status, and more remarkable and statistically significant within subjects with AD‐type dementia. In contrast, arteriolosclerosis, or hemorrhages did not show any inverse association with AD pathologies (Table 3). While their causal relationship is indeed unclear due to the nature of observational data analyses, these results might suggest that widespread impacts of lacunes or infarcts in a long period could be a key to understanding the inverse associations with AD pathologies. Thus, to directly demonstrate the causal effects of cerebrovascular dysfunction on especially tau accumulation, we next introduced a tau propagation model with subsequent BCAS surgery, which provides chronic widespread hypoperfusion due to carotid artery stenosis and could be one of the suitable models to replicate our observations in NACC^24^, ^25^.

### Chronic cerebral hypoperfusion reduces tau pathology in the hippocampus in the Tau/BCAS model mice

**Figure 2:**
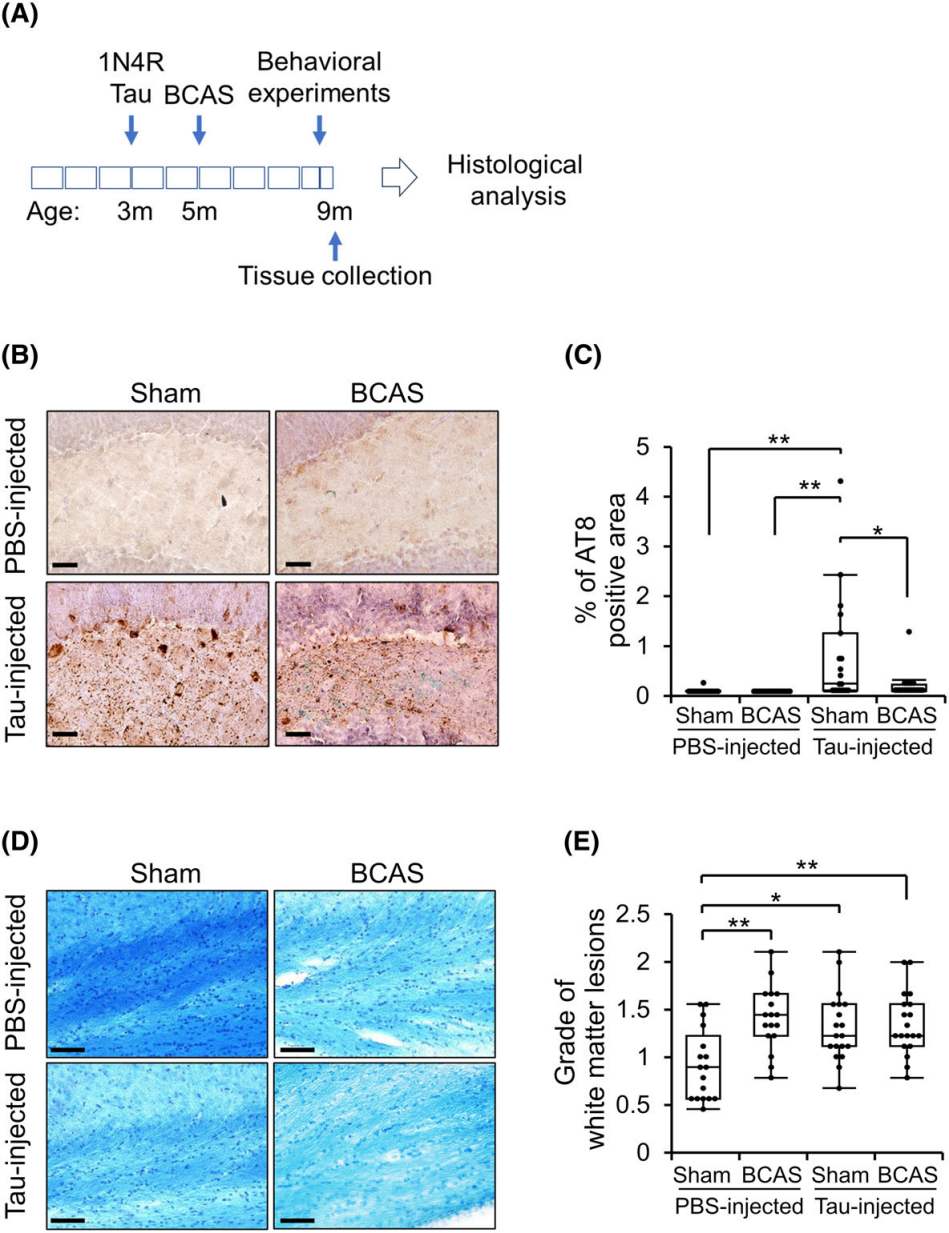
Chronic cerebral hypoperfusion reduced tau accumulation and increased white matter lesions in the Tau/BCAS mice. (A) Experimental schedule. (B) Representative pictures of AT8 staining and (C) percentages of the AT8‐positive area were compared among groups. *P < 0.05 and **P < 0.01, as determined by one‐way ANOVA followed by the Tukey Kramer HSD test (*n* = 15–19 in each group). (D) Klüver‐Barrera staining and (E) grade of white matter lesions were compared among groups. Data are shown as a box‐and‐whisker plot (boxplot) with individual data points. *P < 0.05 and **P < 0.01, as determined by Steel‐Dwass test (*n* = 17–19 in each group). Scale bar = 50 μm.

We conducted a study to evaluate the effect of chronic cerebral hypoperfusion on tau propagation. First, we injected tau seeds, DS‐induced tau assemblies, into the hippocampus of wild‐type mice. After 2 months, we performed BCAS to induce chronic cerebral hypoperfusion. Four months later, we collected brain tissues for histological analysis (Fig. 2A). The immunohistochemical analysis using AT8 antibody, which has been shown to selectively recognize pathological and aggregated tau but not normal human tau,^43^ detected tau phosphorylation at S202/T205 in several brain regions, including the hippocampus, mammillary nucleus, posterior parietal cortex, and entorhinal cortex, suggesting the presence of tau pathology (Fig. S1), as reported in previous study.^23^ In addition, AT8 immunostaining showed co‐localization with Thioflavin S, indicating that the tau detected by AT8 corresponds to aggregated, insoluble tau forms, as previously reported^23^ in this mice model (Fig. S2A). Moreover, PHF1, which recognizes mature tau aggregates^44^ was also co‐localized with anti‐pT212 immunoreactivities, indicating tau in this model is aggregated (Fig. S2B). Quantitative analyses were performed using AT8 antibody on the sections of the injection sites and the hippocampus (2.5 mm posterior to bregma) was analyzed. Interestingly, we found that the tau‐injected BCAS mice showed significantly less AT8‐positive pathology than the tau‐injected Sham mice, indicating suppression of tau propagation or accumulation due to chronic cerebral hypoperfusion (Fig. 2B and C).

Histological analysis of white matter lesions, which include disarrangement of nerve fibers, formation of marked vacuoles, and disappearance of myelinated fibers, characterized by reduced Klüver‐Barrera staining intensity and gliosis, to assess the impact of cerebral hypoperfusion. Consistent with previous findings,^24^, ^35^, ^45^ white matter lesions in the corpus callosum were significantly more severe in the PBS‐injected BCAS group compared to the PBS‐injected Sham group (Fig. 2D and E). In addition, we observed that tau injection itself also increased white matter damage, likely consistent with previous reports, where tau is associated with white matter lesions in humans.^46^, ^47^, ^48^ However, there was no synergic effect of tau injection and BCAS treatment.

### Behavioral characteristics of the Tau/BCAS model mice

**Figure 3:**
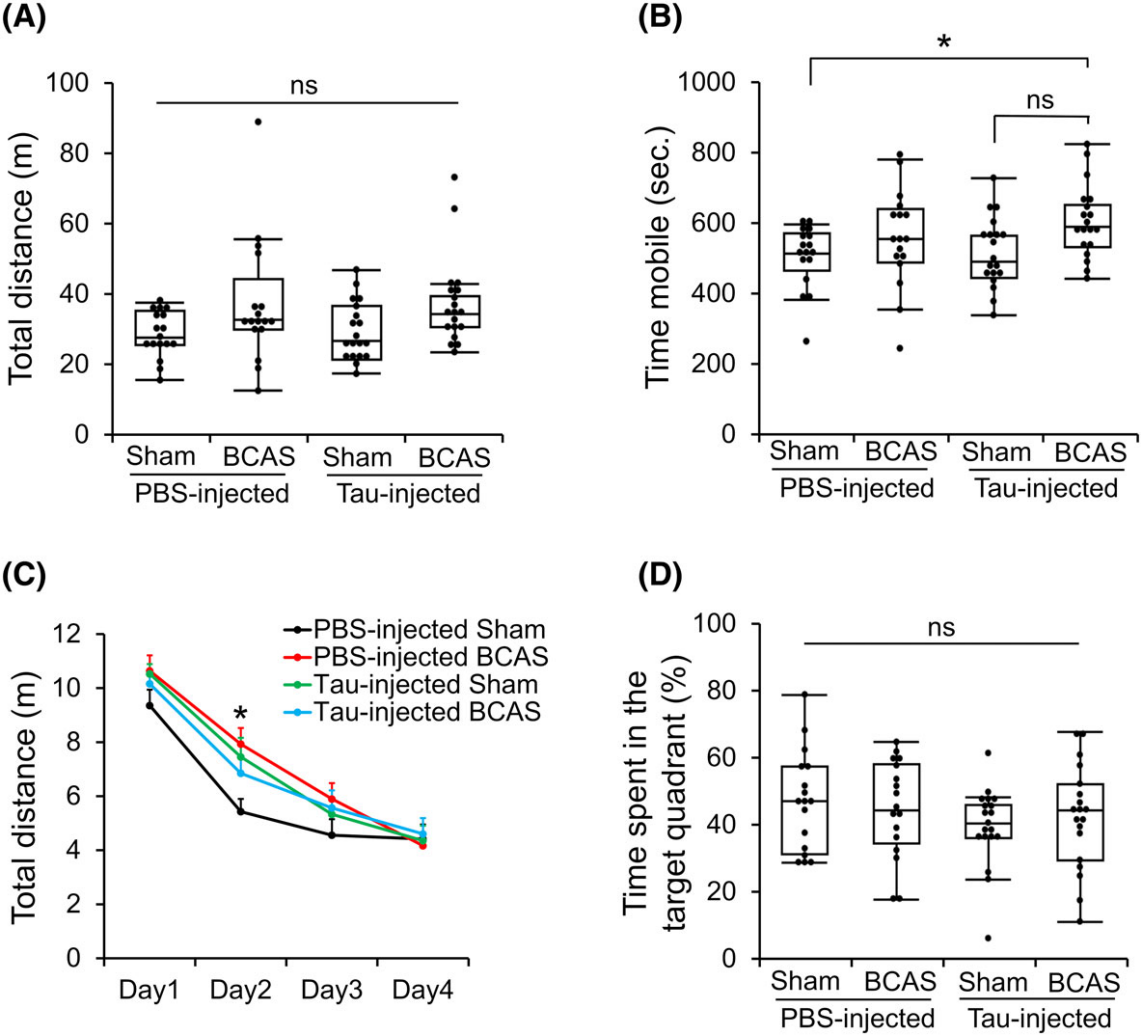
Behavioral characteristics of the Tau/BCAS mice. (A) Total distance traveled and (B) mobile time in the open‐field test. *P < 0.05, by Steel‐Dwass test. (C) Average escape latency during the training period of the Morris water maze test. *P < 0.05 compared with the scores on day 2, as determined by repeated‐measures one‐way ANOVA followed by Tukey HSD test. (D) Time spent in the target quadrant during the probe test. Data are shown as boxplots with individual data points (A, B, D), or as means ± standard error of the mean (C) (*n* = 17–19 in each group). ns, not significant.

To assess the effect of tau injection and chronic cerebral hypoperfusion on the behavior, we performed the open‐field and Morris water maze tests. The tau‐injected BCAS mice showed significantly higher locomotion activity in the open‐field test, especially in mobility time (*P* < 0.05) when compared to the PBS‐injected Sham mice (Fig. 3A and B). The spatial learning in the water maze test was delayed in the PBS‐injected BCAS, tau‐injected Sham, and tau‐injected BCAS groups compared to the PBS‐injected Sham group, while no difference in spatial memory score among the groups (Fig. 3C and D), consistently with a prior study that demonstrated no discernible changes in locomotor activity or spatial memory by BCAS treatment.^49^ These results indicated that tau injection‐induced pathology and chronic cerebral hypoperfusion cause behavioral changes but less synergic effect.

### Co‐localization of phospho‐tau with neurons and glial cell markers in the Tau/BCAS model mice

**Figure 4:**
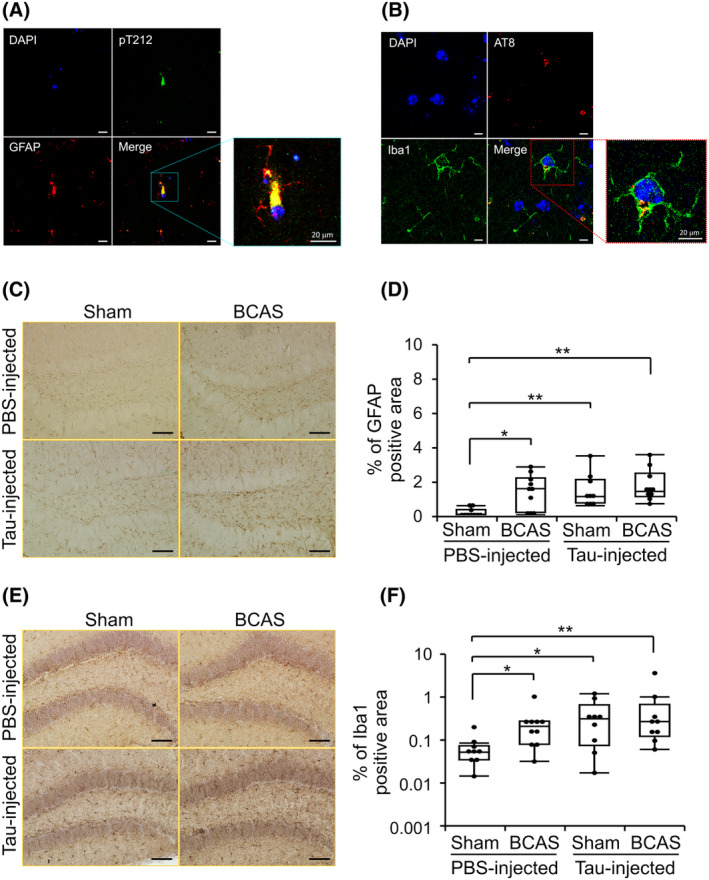
Co‐localization of phospho‐tau and glial markers in the hippocampus and quantification of glial cell marker positive areas in the Tau/BCAS mice. (A) Double staining for GFAP (red) and phospho‐tau (Thr212, green) and (B) phospho‐tau (AT8, red) and Iba1 (green). Scale bar = 20 μm. (C–F) Immunoreactive areas of GFAP (C, D) and Iba1 (E, F) were compared among groups. Data are shown as boxplots with individual data points. *P < 0.05 and **P < 0.01, as determined by Steel‐Dwass test (*n* = 10 in each group). Scale bar = 50 μm.

To verify the cellular location of tau pathology in the brain, we examined immunofluorescence analysis using phospho‐tau antibodies and several cellular markers (neuronal marker, MAP2; mononuclear phagocyte marker, Iba1; astrocyte marker, GFAP; and oligodendrocyte marker, O4). As reported,^23^ abundant tau pathology was detected in neurons (Fig. S3A). Additionally, we observed AT8‐positive signals in astrocytes, microglia, or oligodendrocytes, indicating the presence of tau assemblies in these cells (Fig. 4A and B and Fig. S3B, respectively). These results indicate that tau pathologies were primarily present in neurons, with partial co‐localization in astrocytes, microglial cells, and oligodendrocytes. It is noteworthy that a distinct pattern was observed in the subcellular localization of tau assemblies in astrocytes and microglia. It appears that tau accumulates throughout the cytosol in astrocytes, consistent with previous reports on tau accumulation in astrocytes in neurodegenerative disorders such as CBD and PSP.^50^ On the other hand, tau assemblies seem to be confined to specific compartments in microglia, suggesting engulfment or phagocytosis of tau assemblies by microglia.^51^, ^52^ Quantifying positive areas of GFAP and Iba1 in the hippocampus showed that tau injection or BCAS increased immunoreactivities of those antibodies, consistent with previous reoports,^35^, ^53^ with no significant synergic effect (Fig. 4C–F).

### Chronic cerebral hypoperfusion increased the immunoreactivities of a lysosomal protease, cathepsin D

**Figure 5:**
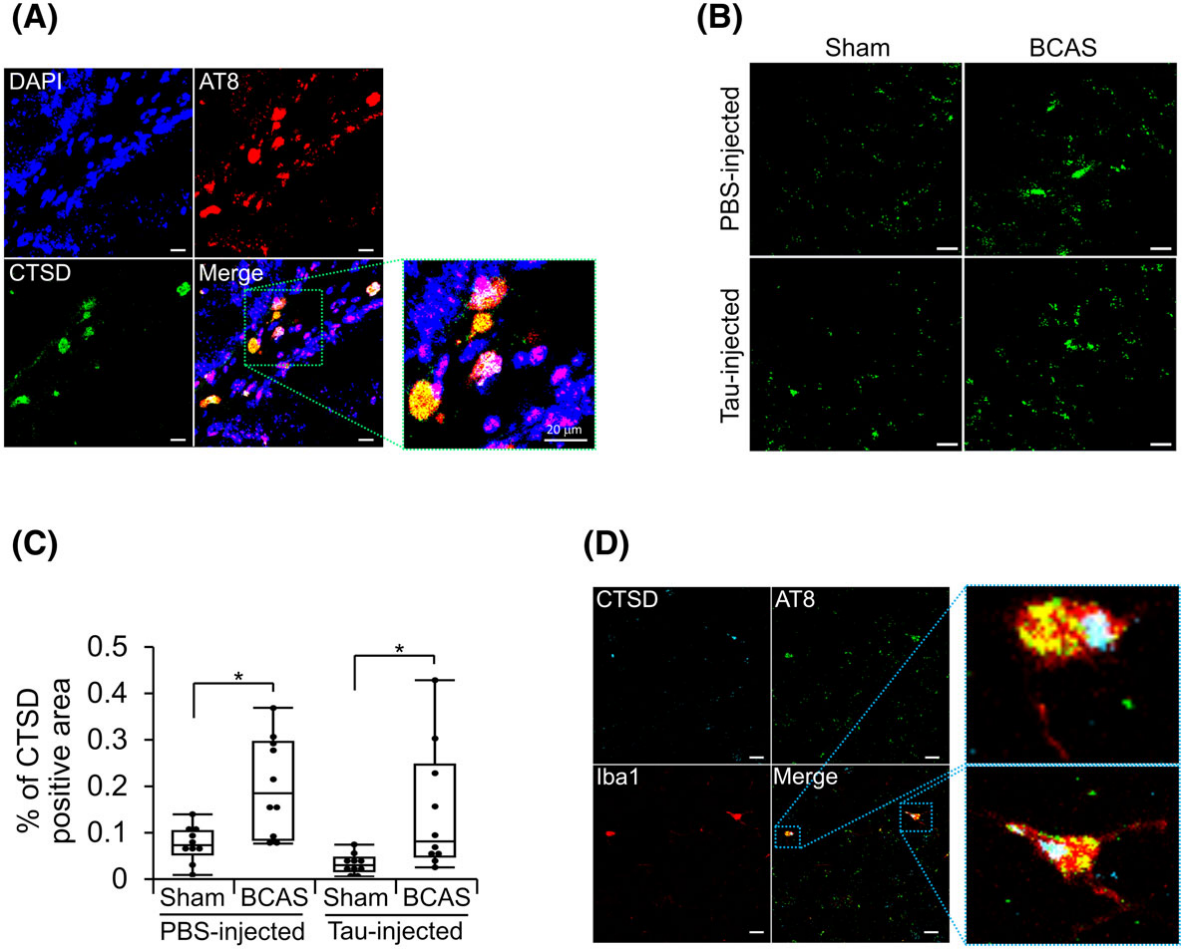
Chronic cerebral hypoperfusion increased immunoreactivities of cathepsin D in the Tau/BCAS mice. (A) Double staining for AT8 (red) and CTSD (green). Scale bar = 20 μm. (B) CTSD immunoreactivities are shown with green fluorescence. Scale bar = 10 μm. (C) Quantified data of CTSD‐positive areas in the hippocampus were compared among groups. (D) Co‐localization of CTSD (cyan), AT8 (green), and Iba1 (red), in the hippocampus. Data are shown as boxplots with individual data points. *P < 0.05, as determined by Steel‐Dwass test (*n* = 10 in each group). Scale bar = 10 μm.

Previous reports indicated that cathepsin D (CTSD), is a lysosomal protease implicated in the clearance of various proteins, including Aβ, tau, and α‐synuclein.^54^, ^55^ CTSD is reported to be upregulated by ischemia in the brain.^56^ To clarify whether the tau degradation process is altered by chronic cerebral hypoperfusion, we performed double staining with phospho‐tau antibodies (AT8, a mouse monoclonal antibody, or phospho‐tau (Thr 212) rabbit polyclonal antibodies, which were used in some immunofluorescence staining instead of AT8), lysosomal‐associated membrane protein 1 (LAMP1), or CTSD antibodies in the hippocampus. We observed that there were overlaps between AT8 and CTSD and between CTSD and LAMP1 (Fig. 5A and Fig. S4, respectively), indicating that the lysosomal CTSD might be involved in tau degradation. Moreover, quantifying CTSD immunoreactivities showed upregulation of CTSD by BCAS (Fig. 5B and C). We also observed co‐localization of AT8, CTSD, and Iba1, suggesting that microglial CTSD involved in tau degradation (Fig. 5D). These findings imply that microglia activated by tau injection and BCAS may uptake tau, which is then degraded by CTSD which is upregulated by BCAS. This may be one mechanism by which BCAS reduces tau pathology.

## Discussions

We obtained novel findings about relationships between tau pathology and cerebrovascular events. First, our analysis of clinical and neuropathological data reveals an inverse association between tau pathology and cerebrovascular events, including stroke and the presence of lacunar infarcts. Second, consistently, we found a reduction in tau pathology by chronic cerebral hypoperfusion in animal models. Furthermore, we observed that cerebral hypoperfusion or tau injection activated microglia, and cerebral hypoperfusion also led to the activation of cathepsin D. Collectively, this study indicates that cerebral hypoperfusion suppressed tau accumulation and that the mechanism might involve an increase in microglial tau phagocytic activity and an elevation in degradation through cathepsin D.

It is not clear how cerebrovascular diseases influence tau accumulation. Studies using tau PET reported that cerebral hypoperfusion or ischemic stroke is positively associated with tau deposition in ischemic brain diseases.^57^, ^58^ There is also a report using animal models, where tau accumulation is increased in tau transgenic mice by cerebral hypoperfusion.^59^ Of note, Coomans et al. recently reported that stroke‐related events in the presence of Aβ pathology are associated with attenuated tau accumulation during early AD,^60^ consistent with our main findings. The differences in observational periods (shorter in former studies vs. more extended in latter studies) or subject backgrounds (non‐AD in former studies vs. mostly AD in latter studies) might contribute to discrepancies among these studies. Longitudinal studies with tau PET before and after cerebrovascular events in a large cohort including people with AD and non‐AD spectrum may address this issue. Also, additional neuropathological studies analyzing the relationship between tau/NFT pathology and infarcts/lacunes in each brain section might be helpful to further clarify the effects of vascular pathologies on AD pathology, in addition to white matter degeneration, which can be positively associated with Braak NFT stage.^46^, ^47^, ^48^ At least, a clinical implication of this study is that cerebrovascular lesions should be considered when evaluating tau accumulation by Tau PET in clinical practice. With regards to microbleeds, Coomans et al. also observed that the co‐occurrence of microbleeds and Aβ pathology was associated with greater tau accumulation during early AD^60^. Because lobar microbleeds are linked to CAA, their study is also consistent with our observation that CAA is positively associated with tau pathology (Estimate = 0.37, *P* < 0.0001). We also observed that the frequency of APOE4 status was relatively decreased in the remote stroke group than in the non‐stroke group. In contrast, it is reported that APOE4 accelerates the development of dementia after stroke.^39^, ^40^ This interaction among APOE, stroke, and dementia should be further investigated in humans and animal models, especially for the mechanism.

The mechanism by which cerebral hypoperfusion reduces tau pathology might be a reduction in tau propagation through increased microglial tau phagocytosis and elevated tau degradation by cathepsin D. Tau propagation might be weakened by impaired neuronal connectivity due to cerebral hypoperfusion.^61^ Of note, cathepsin D activation was only observed in the BCAS‐treated group, whereas microglial activation was observed in both the tau injection and BCAS groups. Indeed, tau was co‐localized with cathepsin D in microglia, suggesting cathepsin D‐mediated tau degradation in microglia is enhanced by cerebral hypoperfusion. It is reported that a history of clinical stroke is associated with plasma P‐tau181 and P‐tau217, while association disappears after adjustment of chronic kidney disease.^62^ Thus, measuring potential changes of plasma levels of p‐tau or its degraded products might be useful to study the mechanism of tau reduction in BCAS mice. Nonetheless, to validate our proposed mechanisms, the use of inhibitors of microglial and/or cathepsin D in experimental settings is warranted. There are other critical limitations in this study, including the recombinant 4R tau that we used. Although 3R and 4R tau form NFT, 4R tau is reported to be predominant in pretangles in AD^63^. We highlight the need for future investigations using different tau isoforms, considering the mixture of 3R and 4R tau in AD.^64^ In addition, we will investigate what might occur if a stroke occurs first and tau seeding is introduced afterward, as previously discussed.^65^

In conclusion, our study demonstrated cerebral hypoperfusion reduced tau pathology, associated with microglial activation and cathepsin D upregulation. Cerebrovascular lesions should be considered when evaluating tau accumulation by Tau PET in clinical practice. This study contributes to understanding of the interplay between cerebrovascular diseases and neurodegenerative processes in older people with multimorbidity.

## Funding Information

This work was supported in part by Research Funding for Longevity Sciences from the National Center for Geriatrics and Gerontology (28‐45, 19‐9, 19‐3, 21‐12, and 24‐16 to NS); Grants‐in‐Aid from Japan Promotion of Science; the Japanese Ministry of Education, Culture, Sports, Science, and Technology (MEXT26293167, MEXT15K15272, MEXT17H04154, MEXT21H02844, and MEXT24K02361 to NS); a Takeda Science Foundation Research Encouragement Grant (to NS and MS); a SENSHIN Medical Research Foundation Research Grant (to NS); a Novartis Foundation for Gerontological Research Award (to NS); an Annual Research Award Grant from the Japanese Society of Anti‐aging Medicine (to NS); a Takeda Medical Research Foundation Research Grant; Mitsui Sumitomo Insurance Welfare Foundation Research Grant (to NS); a NACC Junior Investigator Award (to MS); and National Institutes of Health (NIH) grants R37AG027924, RF1AG057181, RF1AG046205 (to GB).

## Author Contributions

N.S. and M.S. contributed to the conception and design of the study; G.G., M.S., M.M.‐S., A.W., A.S., K.K., G.B., H.T., M.H. and N.S. contributed to the acquisition and analysis of data; G.G. M.S. and N.S. contributed to drafting the manuscript and figures. All authors edited and reviewed the final manuscript.

## Conflicts of Interest

The authors declare no conflicts of interest associated with this manuscript.

## Supporting information


Data S1.

## Data Availability

All data generated or analyzed during this study are available from the corresponding author upon reasonable request. The clinical data are available from NACC upon request.

## References

[1] Ballatore C , Lee VM , Trojanowski JQ . Tau‐mediated neurodegeneration in Alzheimer's disease and related disorders. Nat Rev Neurosci. 2007;8(9):663‐672. 17684513 10.1038/nrn2194

[2] Arendt T , Stieler JT , Holzer M . Tau and tauopathies. Brain Res Bull. 2016;126(Pt 3):238‐292. 27615390 10.1016/j.brainresbull.2016.08.018

[3] Binder LI , Frankfurter A , Rebhun LI . The distribution of tau in the mammalian central nervous system. J Cell Biol. 1985;101(4):1371‐1378. 3930508 10.1083/jcb.101.4.1371 PMC2113928

[4] Kempf M , Clement A , Faissner A , Lee G , Brandt R . Tau binds to the distal axon early in development of polarity in a microtubule‐ and microfilament‐dependent manner. J Neurosci. 1996;16(18):5583‐5592. 8795614 10.1523/JNEUROSCI.16-18-05583.1996 PMC6578978

[5] Berriman J , Serpell LC , Oberg KA , Fink AL , Goedert M , Crowther RA . Tau filaments from human brain and from assembly of recombinant protein show cross‐β structure. Proc Natl Acad Sci USA. 2003;100(15):9034‐9038. 12853572 10.1073/pnas.1530287100 PMC166433

[6] Fitzpatrick AWP , Falcon B , He S , et al. Cryo‐EM structures of tau filaments from Alzheimer's disease. Nature. 2017;547(7662):185‐190. 28678775 10.1038/nature23002 PMC5552202

[7] Rajati F , Rajati M , Rasulehvandi R , Kazeminia M . Prevalence of stroke in the elderly: a systematic review and meta‐analysis. Interdisciplinary. Neurosurgery. 2023;32:101746.

[8] Bots ML , Looman SJ , Koudstaal PJ , Hofman A , Hoes AW , Grobbee DE . Prevalence of stroke in the general population. The Rotterdam study. Stroke. 1996;27(9):1499‐1501. 8784119 10.1161/01.str.27.9.1499

[9] Seshadri S , Wolf PA . Lifetime risk of stroke and dementia: current concepts, and estimates from the Framingham study. Lancet Neurol. 2007;6(12):1106‐1114. 18031707 10.1016/S1474-4422(07)70291-0

[10] Sacco RL , Kasner SE , Broderick JP , et al. An updated definition of stroke for the 21st century a statement for healthcare professionals from the American Heart Association/American Stroke Association. Stroke. 2013;44(7):2064‐2089. 23652265 10.1161/STR.0b013e318296aeca PMC11078537

[11] Riekse RG , Leverenz JB , McCormick W , et al. Effect of vascular lesions on cognition in Alzheimer's disease: a community‐based study. J Am Geriatr Soc. 2004;52(9):1442‐1448. 15341544 10.1111/j.1532-5415.2004.52405.x PMC1487184

[12] White L . Brain lesions at autopsy in older Japanese‐American men as related to cognitive impairment and dementia in the final years of life: a summary report from the Honolulu‐Asia aging study. J Alzheimers Dis. 2009;18(3):713‐725. 19661625 10.3233/JAD-2009-1178

[13] Nation DA , Sweeney MD , Montagne A , et al. Blood‐brain barrier breakdown is an early biomarker of human cognitive dysfunction. Nat Med. 2019;25(2):270‐276. 30643288 10.1038/s41591-018-0297-y PMC6367058

[14] Tayler H , Miners JS , Guzel O , MacLachlan R , Love S . Mediators of cerebral hypoperfusion and blood‐brain barrier leakiness in Alzheimer's disease, vascular dementia and mixed dementia. Brain Pathol. 2021;31(4):e12935. 33410232 10.1111/bpa.12935 PMC8412075

[15] McDade E , Kim A , James J , et al. Cerebral perfusion alterations and cerebral amyloid in autosomal dominant Alzheimer disease. Neurology. 2014;83(8):710‐717. 25031286 10.1212/WNL.0000000000000721 PMC4150128

[16] Johnson NA , Jahng GH , Weiner MW , et al. Pattern of cerebral hypoperfusion in Alzheimer disease and mild cognitive impairment measured with arterial spin‐labeling MR imaging: initial experience. Radiology. 2005;234(3):851‐859. 15734937 10.1148/radiol.2343040197 PMC1851934

[17] Ahmadi K , Pereira JB , Berron D , et al. Gray matter hypoperfusion is a late pathological event in the course of Alzheimer's disease. J Cerebr Blood F Met. 2023;43(4):565‐580. 10.1177/0271678X221141139 PMC10063832 36412244

[18] Clavaguera F , Bolmont T , Crowther RA , et al. Transmission and spreading of tauopathy in transgenic mouse brain. Nat Cell Biol. 2009;11(7):909‐913. 19503072 10.1038/ncb1901 PMC2726961

[19] Iba M , Guo JL , McBride JD , Zhang B , Trojanowski JQ , Lee VM . Synthetic tau fibrils mediate transmission of neurofibrillary tangles in a transgenic mouse model of Alzheimer's‐like tauopathy. J Neurosci. 2013;33(3):1024‐1037. 23325240 10.1523/JNEUROSCI.2642-12.2013 PMC3575082

[20] Jackson SJ , Kerridge C , Cooper J , et al. Short fibrils constitute the major species of seed‐competent tau in the brains of mice transgenic for human P301S tau. J Neurosci. 2016;36(3):762‐772. 26791207 10.1523/JNEUROSCI.3542-15.2016 PMC4719013

[21] Lasagna‐Reeves CA , Castillo‐Carranza DL , Sengupta U , et al. Alzheimer brain‐derived tau oligomers propagate pathology from endogenous tau. Sci Rep. 2012;2:700. 23050084 10.1038/srep00700 PMC3463004

[22] Narasimhan S , Guo JL , Changolkar L , et al. Pathological tau strains from human brains recapitulate the diversity of tauopathies in nontransgenic mouse brain. J Neurosci. 2017;37(47):11406‐11423. 29054878 10.1523/JNEUROSCI.1230-17.2017 PMC5700423

[23] Masuda‐Suzukake M , Suzuki G , Hosokawa M , Nonaka T , Goedert M , Hasegawa M . Dextran sulphate‐induced tau assemblies cause endogenous tau aggregation and propagation in wild‐type mice. Brain Commun. 2020;2(2):fcaa091. 33005889 10.1093/braincomms/fcaa091 PMC7519727

[24] Shibata M , Ohtani R , Ihara M , Tomimoto H . White matter lesions and glial activation in a novel mouse model of chronic cerebral hypoperfusion. Stroke. 2004;35(11):2598‐2603. 15472111 10.1161/01.STR.0000143725.19053.60

[25] Ishikawa H , Shindo A , Mizutani A , Tomimoto H , Lo EH , Arai K . A brief overview of a mouse model of cerebral hypoperfusion by bilateral carotid artery stenosis. J Cereb Blood Flow Metab. 2023;43(2_suppl):18‐36. 36883344 10.1177/0271678X231154597 PMC10638994

[26] Weintraub S , Salmon D , Mercaldo N , et al. The Alzheimer's disease centers' uniform data set (UDS): the neuropsychologic test battery. Alzheimer Dis Assoc Disord. 2009;23(2):91‐101. 19474567 10.1097/WAD.0b013e318191c7dd PMC2743984

[27] Besser LM , Kukull WA , Teylan MA , et al. The revised national Alzheimer's coordinating Center's neuropathology form‐available data and new analyses. J Neuropathol Exp Neurol. 2018;77(8):717‐726. 29945202 10.1093/jnen/nly049 PMC6044344

[28] Shinohara M , Tashiro Y , Suzuki K , Fukumori A , Bu G , Sato N . Interaction between APOE genotype and diabetes in cognitive decline. Alzheimers Dement (Amst). 2020;12(1):e12006. 32211501 10.1002/dad2.12006 PMC7085280

[29] Shinohara M , Gheni G , Hitomi J , Bu G , Sato N . APOE genotypes modify the obesity paradox in dementia. J Neurol Neurosurg Psychiatry. 2023;94(9):670‐680. 37414536 10.1136/jnnp-2022-331034 PMC10695687

[30] Braak H , Braak E . Neuropathological stageing of Alzheimer‐related changes. Acta Neuropathol. 1991;82(4):239‐259. 1759558 10.1007/BF00308809

[31] Mirra SS , Heyman A , McKeel D , et al. The consortium to establish a registry for Alzheimer's disease (CERAD). Part II. Standardization of the neuropathologic assessment of Alzheimer's disease. Neurology. 1991;41(4):479‐486. 2011243 10.1212/wnl.41.4.479

[32] Vonsattel JPG , Myers RH , Hedleywhyte ET , Ropper AH , Bird ED , Richardson EP . Cerebral amyloid Angiopathy without and with cerebral hemorrhages—a comparative histological study. Ann Neurol. 1991;30(5):637‐649. 1763890 10.1002/ana.410300503

[33] Taniguchi S , Suzuki N , Masuda M , et al. Inhibition of heparin‐induced tau filament formation by phenothiazines, polyphenols, and porphyrins. J Biol Chem. 2005;280(9):7614‐7623. 15611092 10.1074/jbc.M408714200

[34] Hasegawa M , Smith MJ , Goedert M . Tau proteins with FTDP‐17 mutations have a reduced ability to promote microtubule assembly. FEBS Lett. 1998;437(3):207‐210. 9824291 10.1016/s0014-5793(98)01217-4

[35] Suzuki K , Shinohara M , Uno Y , et al. Deletion of B‐cell translocation gene 2 (BTG2) alters the responses of glial cells in white matter to chronic cerebral hypoperfusion. J Neuroinflammation. 2021;18(1):86. 33812385 10.1186/s12974-021-02135-w PMC8019185

[36] Giacanelli F , Moretti E . On a new technic for the combined staining of nerve cells and fibers: the Kluver‐Barrera method. Riv Patol Nerv Ment. 1959;80:467‐469. 13827791

[37] Wakita H , Tomimoto H , Akiguchi I , Kimura J . Glial activation and white matter changes in the rat brain induced by chronic cerebral hypoperfusion: an immunohistochemical study. Acta Neuropathol. 1994;87(5):484‐492. 8059601 10.1007/BF00294175

[38] Sudlow C , Martinez Gonzalez NA , Kim J , Clark C . Does apolipoprotein E genotype influence the risk of ischemic stroke, intracerebral hemorrhage, or subarachnoid hemorrhage? Systematic review and meta‐analyses of 31 studies among 5961 cases and 17,965 controls. Stroke. 2006;37(2):364‐370. 16385096 10.1161/01.STR.0000199065.12908.62 PMC2577180

[39] Pendlebury ST , Poole D , Burgess A , Duerden J , Rothwell PM , Oxford VS . APOE‐epsilon4 genotype and dementia before and after transient ischemic attack and stroke: population‐based cohort study. Stroke. 2020;51(3):751‐758. 32070224 10.1161/STROKEAHA.119.026927 PMC7224982

[40] Montagne A , Nation DA , Zlokovic BV . APOE4 accelerates development of dementia after stroke: is there a role for cerebrovascular dysfunction? Stroke. 2020;51(3):699‐700. 32070225 10.1161/STROKEAHA.119.028814 PMC7041876

[41] Martinez‐Gonzalez NA , Sudlow CL . Effects of apolipoprotein E genotype on outcome after ischaemic stroke, intracerebral haemorrhage and subarachnoid haemorrhage. J Neurol Neurosurg Psychiatry. 2006;77(12):1329‐1335. 16926234 10.1136/jnnp.2006.097543 PMC2077401

[42] Okamoto Y , Yamamoto T , Kalaria RN , et al. Cerebral hypoperfusion accelerates cerebral amyloid angiopathy and promotes cortical microinfarcts. Acta Neuropathol. 2012;123(3):381‐394. 22170742 10.1007/s00401-011-0925-9 PMC3282897

[43] Mercken M , Vandermeeren M , Lubke U , et al. Monoclonal‐antibodies with selective specificity for Alzheimer tau are directed against phosphatase‐sensitive epitopes. Acta Neuropathol. 1992;84(3):265‐272. 1384266 10.1007/BF00227819

[44] Greenberg SG , Davies P , Schein JD , Binder LI . Hydrofluoric acid‐treated tau PHF proteins display the same biochemical properties as normal tau. J Biol Chem. 1992;267(1):564‐569. 1370450

[45] Shindo A , Liang AC , Maki T , et al. Subcortical ischemic vascular disease: roles of oligodendrocyte function in experimental models of subcortical white‐matter injury. J Cereb Blood Flow Metab. 2016;36(1):187‐198. 25920960 10.1038/jcbfm.2015.80 PMC4758561

[46] Strain JF , Smith RX , Beaumont H , et al. Loss of white matter integrity reflects tau accumulation in Alzheimer disease defined regions. Neurology. 2018;91(4):e313‐e8. 29959265 10.1212/WNL.0000000000005864 PMC6070383

[47] Pereira JB , Ossenkoppele R , Palmqvist S , et al. Amyloid and tau accumulate across distinct spatial networks and are differentially associated with brain connectivity. elife. 2019;8:e50830. 31815669 10.7554/eLife.50830 PMC6938400

[48] Polvikoski TM , van Straaten ECW , Barkhof F , et al. Frontal lobe white matter hyperintensities and neurofibrillary pathology in the oldest old. Neurology. 2010;75(23):2071‐2078. 21048201 10.1212/WNL.0b013e318200d6f9 PMC2995533

[49] Shibata M , Yamasaki N , Miyakawa T , et al. Selective impairment of working memory in a mouse model of chronic cerebral hypoperfusion. Stroke. 2007;38(10):2826‐2832. 17761909 10.1161/STROKEAHA.107.490151

[50] Feany MB , Dickson DW . Neurodegenerative disorders with extensive tau pathology: a comparative study and review. Ann Neurol. 1996;40(2):139‐148. 8773594 10.1002/ana.410400204

[51] Majerova P , Zilkova M , Kazmerova Z , et al. Microglia display modest phagocytic capacity for extracellular tau oligomers. J Neuroinflammation. 2014;11:161. 25217135 10.1186/s12974-014-0161-z PMC4172893

[52] Das R , Balmik AA , Chinnathambi S . Phagocytosis of full‐length tau oligomers by actin‐remodeling of activated microglia. J Neuroinflammation. 2020;17(1):10. 31915009 10.1186/s12974-019-1694-y PMC6950897

[53] Dutta D , Jana M , Paidi RK , et al. Tau fibrils induce glial inflammation and neuropathology via TLR2 in Alzheimer's disease‐related mouse models. J Clin Invest. 2023;133(18):e161987. 37552543 10.1172/JCI161987 PMC10503811

[54] Kenessey A , Nacharaju P , Ko LW , Yen SH . Degradation of tau by lysosomal enzyme cathepsin D: implication for Alzheimer neurofibrillary degeneration. J Neurochem. 1997;69(5):2026‐2038. 9349548 10.1046/j.1471-4159.1997.69052026.x

[55] Bednarski E , Lynch G . Cytosolic proteolysis of tau by cathepsin D in hippocampus following suppression of cathepsins B and L. J Neurochem. 1996;67(5):1846‐1855. 8863489 10.1046/j.1471-4159.1996.67051846.x

[56] Nakanishi H , Tsukuba T , Kondou T , Tanaka T , Yamamoto K . Transient forebrain ischemia induces increased expression and specific localization of cathepsins E and D in rat hippocampus and neostriatum. Exp Neurol. 1993;121(2):215‐223. 8339772 10.1006/exnr.1993.1088

[57] Yamauchi H , Kagawa S , Kusano K , Ito M , Okuyama C . Misery perfusion and tau deposition in atherosclerotic major cerebral artery disease: a (18)F‐Florzolotau positron emission tomography study. Stroke. 2022;53(12):e500‐e3. 36337055 10.1161/STROKEAHA.122.040493 PMC9698085

[58] Michiels L , Thijs L , Mertens N , et al. In vivo detection of neurofibrillary tangles by (18)F‐MK‐6240 PET/MR in patients with ischemic stroke. Neurology. 2023;100(1):e62‐e71. 36302665 10.1212/WNL.0000000000201344

[59] Shimada T , Shindo A , Matsuyama H , et al. Chronic cerebral hypoperfusion upregulates leptin receptor expression in astrocytes and tau phosphorylation in tau transgenic mice. Neurosci Lett. 2019;704:133‐140. 30954605 10.1016/j.neulet.2019.04.009

[60] Coomans EM , van Westen D , Pichet Binette A , et al. Interactions between vascular burden and amyloid‐beta pathology on trajectories of tau accumulation. Brain. 2024;147(3):949‐960. 37721482 10.1093/brain/awad317 PMC10907085

[61] Vogel JW , Iturria‐Medina Y , Strandberg OT , et al. Spread of pathological tau proteins through communicating neurons in human Alzheimer's disease. Nat Commun. 2020;11(1):2612. 32457389 10.1038/s41467-020-15701-2 PMC7251068

[62] Mielke MM , Dage JL , Frank RD , et al. Performance of plasma phosphorylated tau 181 and 217 in the community. Nat Med. 2022;28(7):1398‐1405. 35618838 10.1038/s41591-022-01822-2 PMC9329262

[63] Hara M , Hirokawa K , Kamei S , Uchihara T . Isoform transition from four‐repeat to three‐repeat tau underlies dendrosomatic and regional progression of neurofibrillary pathology. Acta Neuropathol. 2013;125(4):565‐579. 23407988 10.1007/s00401-013-1097-6

[64] Hosokawa M , Masuda‐Suzukake M , Shitara H , et al. Development of a novel tau propagation mouse model endogenously expressing 3 and 4 repeat tau isoforms. Brain. 2022;145(1):349‐361. 34515757 10.1093/brain/awab289

[65] Mukaetova‐Ladinska EB , Abdel‐All Z , Mugica ES , et al. Tau proteins in the temporal and frontal cortices in patients with vascular dementia. J Neuropathol Exp Neurol. 2015;74(2):148‐157. 25575131 10.1097/NEN.0000000000000157

