# Supplementary material for: Cerebral hypoperfusion reduces tau accumulation
Source: Ann Clin Transl Neurol. 2024 Dec 2;12(1):69–85. doi: 10.1002/acn3.52247 (PMC11752094; doi:10.1002/acn3.52247)
Supplement: Supplementary file 1 — Data S1. [file ACN3-12-69-s001.pdf]

Supplementary materials

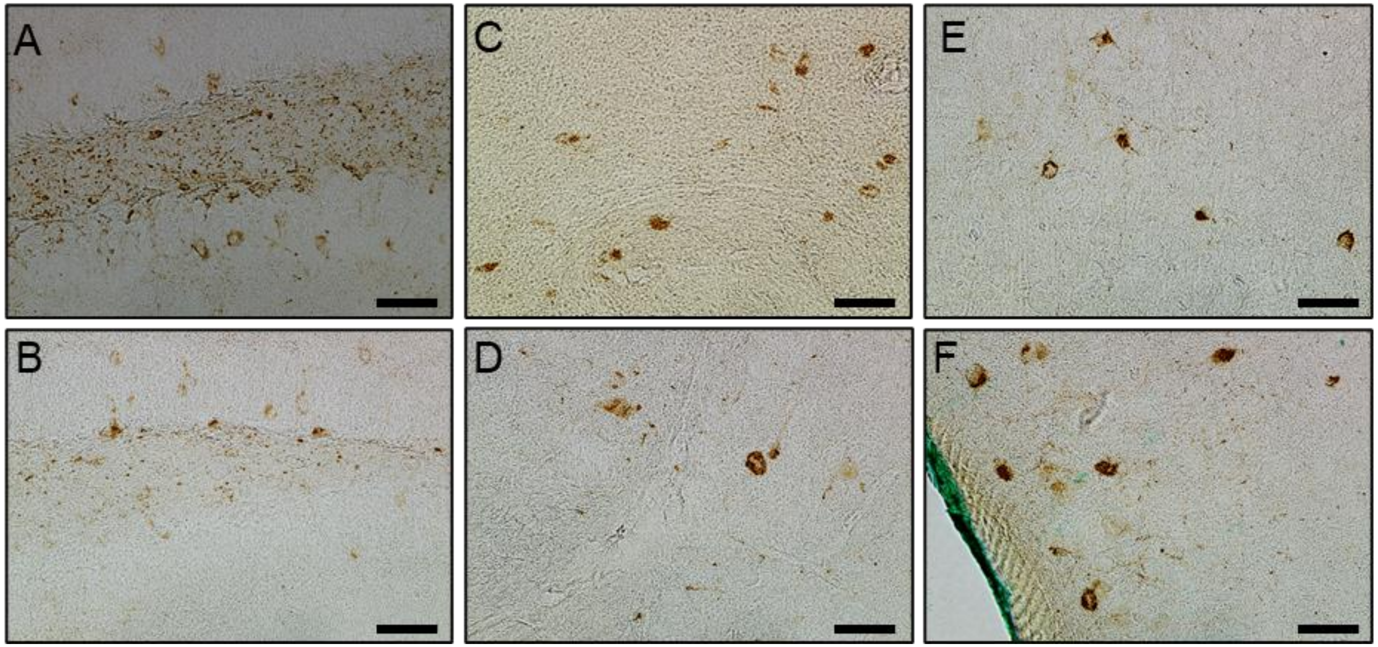

Supplementary Figure 1. AT8 staining in tau-injected mice. Phosphorylated (S202/T205) tau was detected in several brain regions, at the injection site (ipsilateral) and contralateral site. (A, B) Hippocampus, (C, D) mammillary nucleus, (E) posterior parietal cortex, (F) entorhinal cortex, (A, C, E) ipsilateral, (B, D, F) contralateral. Scale bar = 50 μm.

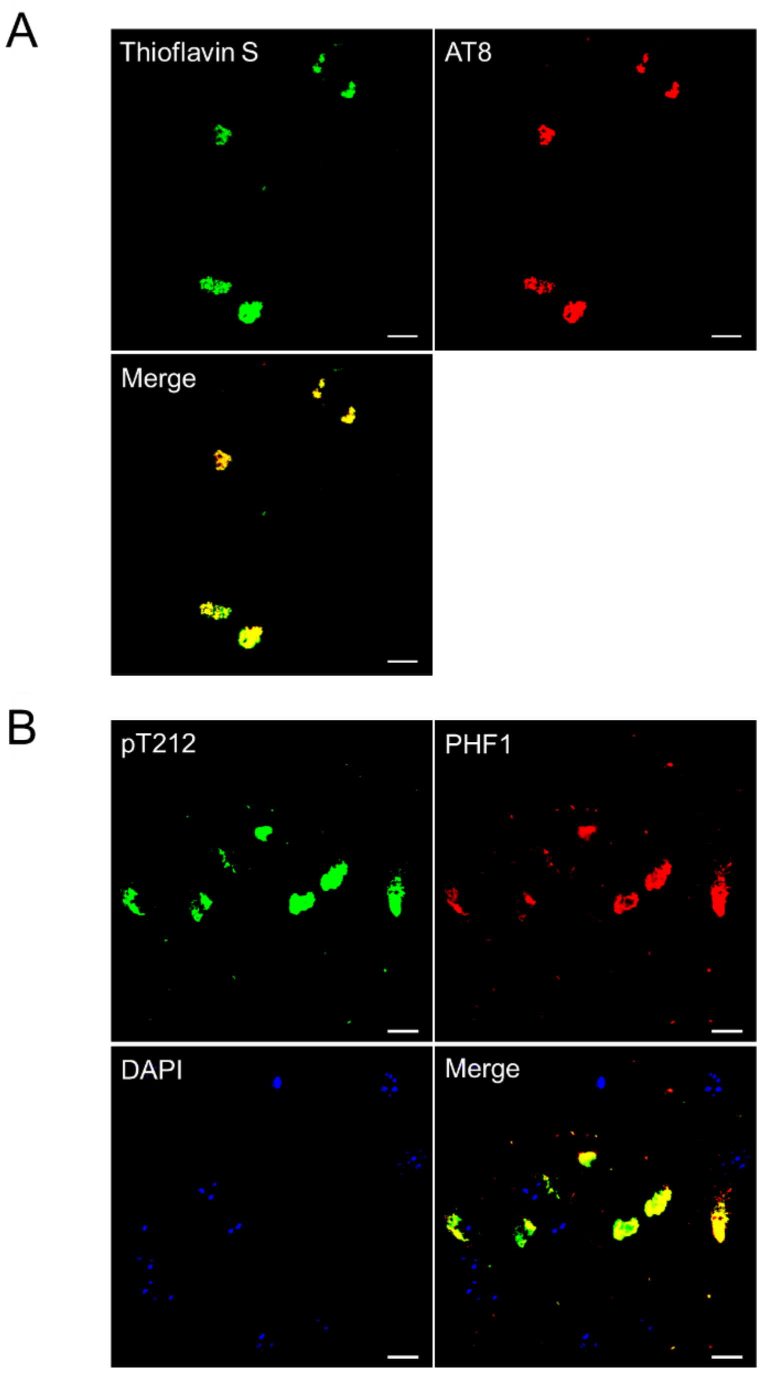

Supplementary Figure 2. Double staining of the brain sections with anti-phospho-tau antibodies and Thioflavin S or PHF1 antibody.

(A) Double immunofluorescence with anti-phospho-tau antibody, AT8 (red) and Thioflavin S (green), (B) Double immunofluorescence with PHF1 antibody (red) and anti-phospho-tau pT212 antibody (green) in tau-injected mice. Scale bar = 10 μm.

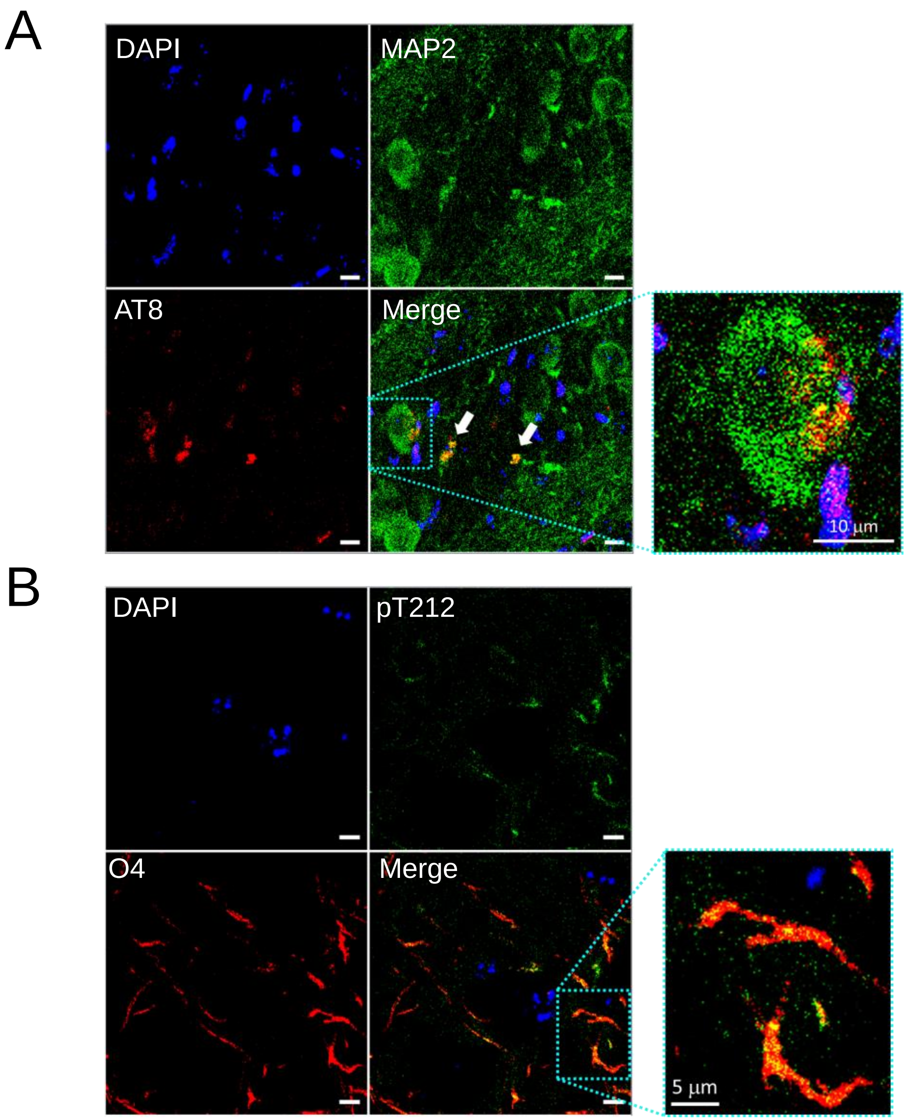

Supplementary Figure 3. Double staining of the brain sections with phospho-tau (AT8 or pT212) and neuronal marker MAP2 or oligodendrocyte O4 antibodies.

(A) Double immunofluorescence with AT8 (red) and anti-MAP2 (green), White arrows show colocalization. Scale bar = 10 µm. (B) Double immunofluorescence with O4 (red) and phospho-tau (Thr212) (green) in tau-injected mice. DAPI, 4'6-Diamidino-2-Phenylindole (nuclear staining dye). Scale bar = 5 µm.

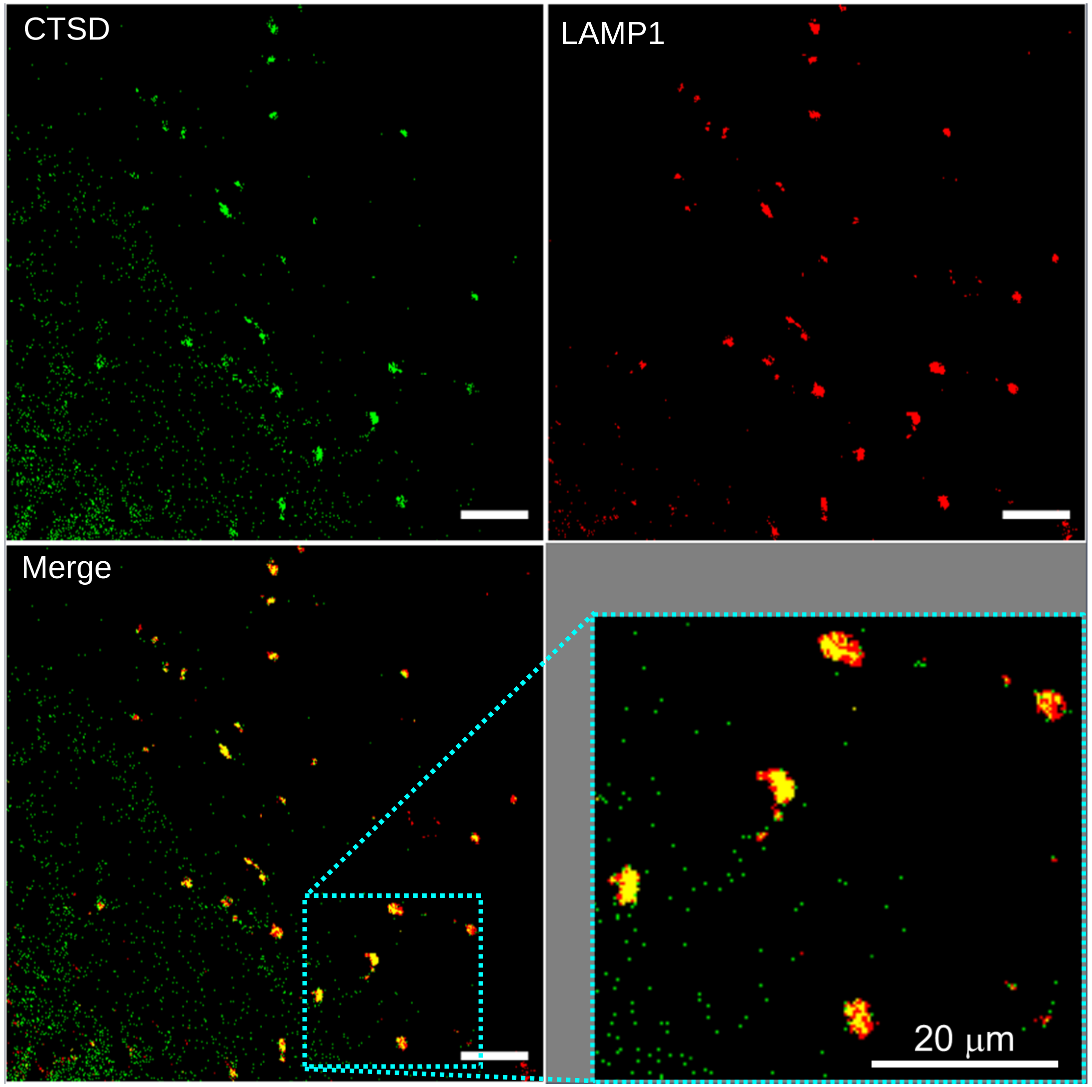

Supplementary Figure 4. Double staining of the brain sections with lysosomal protein LAMP1 and CTSD antibodies.

Double immunofluorescence with LAMP1 (red), and CTSD (green) in tau-injected mice. Scale bar 20 μm. DAPI, 4'6-Diamidino-2-Phenylindole (nuclear staining dye); LAMP1, lysosomal-associated membrane protein 1; CTSD, cathepsin D.

Supplementary Table 1. Effects of vascular pathologies on Alzheimer's pathologies in the model using sex, age, race, APOE4, and cognitive/dementia status as co-variants.

Effects of vascular pathologies	On diffuse plaques		On neuritic plaques		On Braak NFT		On CAA	
	Estimate*	*p*-value	Estimate*	*p*-value	Estimate*	*p*-value	Estimate*	*p*-value
Arteriolosclerosis (0=none,1=mild, 2=moderate, 3=severe)	0.04 (0.01, 0.08)	0.021	0.04 (0.01, 0.08)	0.0218	0.07 (0.01, 0.12)	0.0258	0.15 (0.12, 0.19)	<0.0001
Atherosclerosis of the circle of Willis (0=none,1=mild, 2=moderate, 3=severe)	0.08 (0.04, 0.11)	<0.0001	0.08 (0.05, 0.12)	<0.0001	0.1 (0.04, 0.16)	0.0005	0.06 (0.02, 0.09)	0.0005
Ischemic, hemorrhagic, or vascular pathology present (0=no, 1=one or more vascular pathology)	0.52 (0.28, 0.76)	<0.0001	0.49 (0.26, 0.72)	<0.0001	1.01 (0.64, 1.37)	<0.0001	0.82 (0.61, 1.03)	<0.0001
Large arterial infarcts present (0=no, 1=yes)	-0.14 (-0.28, 0.01)	0.0598	-0.18 (-0.32, -0.05)	0.0076	-0.12 (-0.33, 0.09)	0.2615	0.19 (0.07, 0.32)	0.002
Infarcts and lacunes (0=no, 1=yes)	-0.09 (-0.18, -0.01)	0.0215	-0.08 (-0.16, 0)	0.0446	-0.19 (-0.32, -0.07)	0.0026	0.09 (0.02, 0.16)	0.0161
One or more lacunes (0=no, 1=yes)	-0.1 (-0.21, 0.01)	0.0827	-0.1 (-0.2, 0.01)	0.0783	-0.16 (-0.33, 0.01)	0.064	0 (-0.1, 0.1)	0.987
Single or multiple hemorrhages present (0=no, 1=yes)	0.08 (-0.1, 0.26)	0.3623	0.11 (-0.06, 0.28)	0.1957	-0.06 (-0.33, 0.2)	0.6566	0.24 (0.09, 0.39)	0.0022
Multiple microinfarcts present (0=no, 1=yes)	0.02 (-0.1, 0.13)	0.7869	-0.04 (-0.15, 0.06)	0.8539	-0.02 (-0.19, 0.15)	0.5195	0.08 (-0.01, 0.18)	0.0967
Microinfarcts (0=no, 1 =yes)	0.03 (-0.05, 0.11)	0.4887	0.01 (-0.07, 0.09)	0.4045	0.04 (-0.08, 0.16)	0.8067	0.1 (0.03, 0.17)	0.0053
White matter rarefaction (0=none,1=mild, 2=moderate, 3=severe)	0.03 (-0.02, 0.09)	0.1772	0.06 (0.01, 0.11)	0.0299	0 (-0.08, 0.09)	0.9528	0.11 (0.06, 0.16)	<0.0001
Subcortical arteriosclerotic leukoencephalopathy present (0=no, 1=yes)	-0.16 (-0.28, -0.05)	0.0062	-0.16 (-0.27, -0.05)	0.0056	0.11 (-0.07, 0.29)	0.2166	0 (-0.1, 0.1)	0.9987

*Estimate with 95% confidence interval.

The blue color indicates the negative effects with statistical significance, while the red color indicates the positive effects with statistical significance.

Supplementary Table 2. Old infarcts observed at pathological assessments of subjects with or without remote stroke history in the NACC database

	Total (includes recent stroke history)	Non-stroke	Remote stroke history	*p*-value
Old infarcts observed grossly (whole areas, including lacunes) (no: yes)	1564: 260	1469: 196	95: 64	<0.001
In cerebral cortex				
Any observed (no: yes)	1704: 108	1584: 74	120: 34	<0.001
Number of old infarcts	0.10±0.46	0.07±0.39	0.39±0.83	<0.001
Size of largest old infarct (cm)	0.20±1.04	0.12±0.77	1.12±2.45	<0.001
Size of second-largest old infarct (cm)	0.04±0.33	0.02±0.24	0.30±0.87	<0.001
In subcortical/periventricular white matter				
Any observed (no: yes)	1757: 53	1617: 40	140: 13	<0.001
Number of old infarcts	0.04±0.31	0.04±0.29	0.12±0.45	<0.001
Size of largest old infarct (cm)	0.05±0.38	0.03±0.29	0.24±0.97	<0.001
Size of second-largest old infarct (cm)	0.00±0.07	0.00±0.06	0.04±0.19	<0.001
In deep cerebral gray matter or internal capsule				
Any observed (no: yes)	1714: 96	1585: 71	129: 25	<0.001
Number of old infarcts	0.08±0.46	0.06±0.38	0.31±0.96	<0.001
Size of largest old infarct (cm)	0.07±0.37	0.05±0.31	0.28±0.76	<0.001
Size of second-largest old infarct (cm)	0.01±0.10	0.01±0.08	0.06±0.25	<0.001
In brainstem or cerebellum				
Any observed (no: yes)	1761: 48	1623: 33	138: 15	<0.001
Number of old infarcts	0.03±0.22	0.02±0.16	0.15±0.53	<0.001
Size of largest old infarct (cm)	0.05±0.50	0.04±0.48	0.20±0.67	<0.001
Size of second-largest old infarct (cm)	0.00±0.08	0.00±0.05	0.05±0.25	<0.001

For continuous data, values are the mean ± SD.

P-values are from one-way ANOVA (continuous data) or the chi-square test (categorical value).

Regarding number/size of old infarcts in each brain areas, we evaluated following variables, “NPINF” (Old infarcts observed grossly (including lacunes); 0=no,1=yes), “NPINF1A” (Old infarcts observed grossly — number in cerebral cortex), “NPINF1B” (Size of largest old infarct observed in cerebral cortex (cm)), “NPINF1D” (Size of second largest old infarct observed in cerebral cortex (cm)), “NPINF2A” (Old infarcts observed grossly — number in subcortical cerebral/periventricular white matter), “NPINF2B” (Size of largest old infarct observed in subcortical cerebral/periventricular white matter (cm)), “NPINF2D” (Size of second largest old infarct observed in subcortical cerebral/periventricular white matter (cm)), “NPINF3A” (Old infarcts observed grossly — number in deep cerebral gray matter or internal capsule), “NPINF3B” (Size of largest old infarct observed in deep cerebral gray matter or internal capsule (cm)), “NPINF3D” (Size of second-largest old infarct observed in deep cerebral gray matter or internal capsule (cm)), “NPINF4A” (Old infarcts observed grossly — number in brainstem or cerebellum), “NPINF4B” (Size of largest old infarct observed in brainstem or cerebellum (cm)), and “NPINF4D” (Size of second-largest old infarct observed in brainstem or cerebellum (cm)), after removing subjects whose vascular pathology scores were missing or unavailable.

Supplementary Table 3. Effects of number/size of old infarcts in each area on Alzheimer's pathologies in the model include sex, age, race, and APOE4 as co-variants within AD-type dementia.

Effects of old infarcts	On diffuse plaques		On neuritic plaques		On Braak NFT		On CAA	
	Estimate*	*p*-value	Estimate*	*p*-value	Estimate*	*p*-value	Estimate*	*p*-value
Old infarcts observed grossly (whole areas, including lacunes) (0=no,1=yes)	-0.02 (-0.16, 0.12)	0.7613	-0.07 (-0.22, 0.07)	0.3049	-0.28 (-0.5, -0.07)	0.0109	0.23 (0.06, 0.4)	0.0076
In cerebral cortex								
Any observed (0=no,1=yes)	-0.05 (-0.26, 0.15)	0.6135	-0.1 (-0.31, 0.11)	0.3573	-0.34 (-0.66, -0.02)	0.0402	0.38 (0.13, 0.63)	0.0028
Number of old infarcts	-0.02 (-0.12, 0.09)	0.7407	-0.03 (-0.14, 0.07)	0.5411	-0.16 (-0.32, 0.01)	0.0578	0.13 (0, 0.25)	0.0439
Size of largest old infarct	-0.02 (-0.07, 0.03)	0.432	-0.04 (-0.09, 0.01)	0.1048	-0.07 (-0.14, 0.01)	0.0925	0.08 (0.02, 0.14)	0.0096
Size of second-largest old infarct	-0.05 (-0.19, 0.09)	0.4803	-0.08 (-0.23, 0.06)	0.2558	-0.26 (-0.48, -0.04)	0.0213	0.16 (-0.01, 0.33)	0.0602
In subcortical/periventricular white matter								
Any observed (1=yes, 0=no)	0.02 (-0.26, 0.3)	0.877	0.05 (-0.23, 0.33)	0.7207	-0.16 (-0.59, 0.27)	0.4601	-0.07 (-0.4, 0.26)	0.6853
Number of old infarcts	0.03 (-0.11, 0.17)	0.6914	0.04 (-0.11, 0.18)	0.6063	-0.1 (-0.32, 0.12)	0.3807	0.03 (-0.14, 0.2)	0.7512
Size of largest old infarct	0.02 (-0.12, 0.16)	0.7671	0.05 (-0.1, 0.2)	0.5352	0.05 (-0.17, 0.28)	0.6597	0.06 (-0.12, 0.23)	0.5214
Size of second-largest old infarct	0.13 (-0.56, 0.82)	0.7135	0.12 (-0.55, 0.79)	0.7183	-0.54 (-1.55, 0.48)	0.2989	0.27 (-0.52, 1.05)	0.502
In deep cerebral gray matter or internal capsule								

Any observed (1=yes, 0=no)	-0.15 (-0.37, 0.06)	0.1648	-0.23 (-0.45, -0.02)	0.0353	-0.36 (-0.7, -0.03)	0.0335	0.14 (-0.12, 0.39)	0.2981
Number of old infarcts	-0.08 (-0.17, 0.01)	0.0958	-0.11 (-0.2, -0.01)	0.032	-0.14 (-0.29, 0.01)	0.0733	0.01 (-0.11, 0.12)	0.9224
Size of largest old infarct	-0.74 (-1.23, -0.26)	0.0026	-0.8 (-1.31, -0.29)	0.0022	-0.46 (-1.24, 0.33)	0.2533	0.21 (-0.38, 0.81)	0.4777
Size of second-largest old infarct	-0.74 (-1.23, -0.26)	0.0026	-0.8 (-1.31, -0.29)	0.0022	-0.46 (-1.24, 0.33)	0.2533	0.21 (-0.38, 0.81)	0.4777
In brainstem or cerebellum								
Any observed (1=yes, 0=no)	-0.03 (-0.34, 0.28)	0.842	-0.12 (-0.43, 0.19)	0.4523	-0.53 (-1.01, -0.05)	0.0298	0.16 (-0.2, 0.52)	0.3896
Number of old infarcts	-0.05 (-0.32, 0.22)	0.7168	-0.14 (-0.41, 0.12)	0.2782	-0.5 (-0.9, -0.1)	0.0149	0.09 (-0.22, 0.39)	0.5793
Size of largest old infarct	-0.04 (-0.2, 0.11)	0.5866	-0.09 (-0.25, 0.07)	0.2609	-0.25 (-0.51, 0)	0.0534	0.11 (-0.07, 0.3)	0.2314
Size of second-largest old infarct	-0.11 (-1.2, 0.98)	0.8427	-0.41 (-1.35, 0.54)	0.3993	-1.4 (-2.82, 0.01)	0.0521	-0.12 (-1.22, 0.98)	0.8347

*Estimate with 95% confidence interval.

The blue color indicates the negative effects with statistical significance, while the red color indicates the positive effec ts with statistical significance. Regarding number/size of old infarcts in each brain areas, we evaluated following variables, "NPINF" (Old infarcts observed grossly (including lacunes); 0=no,1=yes), "NPINF1A" (Old infarcts observed grossly — number in cerebral cortex), "NPINF1B" (Size of largest old infarct observed in cerebral cortex (cm)), "NPINF1D" (Size of second largest old infarct observed in cerebral cortex (cm)), "NPINF2A" (Old infarcts observed grossly — number in subcortical cerebral/periventricular white matter), "NPINF2B" (Size of largest old infarct observed in subcortical cerebral/periventricular white matter (cm)), "NPINF2D" (Size of second largest old infarct observed in subcortical cerebral/periventricular white matter (cm)), "NPINF3A" (Old infarcts observed grossly — number in deep cerebral gray matter or internal capsule), "NPINF3B" (Size of largest old infarct observed in deep cerebral gray matter or internal capsule (cm)), "NPINF3D" (Size of second-largest old infarct observed in deep cerebral gray matter or internal capsule (cm)), "NPINF4A" (Old infarcts observed grossly — number in brainstem or cerebellum), "NPINF4B" (Size of largest old infarct observed in brainstem or cerebellum (cm)), and "NPINF4D" (Size of second-largest old infarct observed in brainstem or cerebellum (cm)), after removing subjects whose vascular pathology scores were missing or unavailable.

Supplementary Table 4. List of primary antibodies used in this study.

Primary antibody	Host	Supplier	Cat#	Dilution
AT8 (Phospho-tau at Ser202/Thr205)	Ms monoclonal	Thermo Scientific	NA163653	IHC: 1/1000; IF: 1/200
Phospho-tau (Thr212)	Rb polyclonal	Invitrogen	44740G	IF: 1/200
PHF1 (Phospho-tau at Ser396/Ser404)	Ms monoclonal	Provided by Dr. Peter Davies		IF: 1/5
MAP2	Rb polyclonal	Abcam	ab183830	IF: 1/500
GFAP	Rb polyclonal	Sigma	G9269	IF: 1/500
GFAP	Ms monoclonal	Proteintech	60190-1-1g	IF: 1/500
Iba1	Rb polyclonal	Wako	019-19741	IF: 1/100
Iba1	Goat polyclonal	Abcam	Ab5076	IF: 1/100
O4	Ms monoclonal	R&D systems	MAB1326	IF: 1/100
Olig2	Ms monoclonal	Millipore	MABN50	IF: 1/100
LAMP1	Ms monoclonal	BD Biosciences	51-9002014	IF: 1/200
CTSD	Rb monoclonal	Abcam	EPR 3056Y	IF: 1/200

PHF1: paired helical filaments 1; MAP2: microtubule associated protein 2; GFAP: glial fibrillary acidic protein; Iba1: ionized calcium-binding adapter molecule 1; LAMP1: lysosomal-associated membrane protein 1; CTSD: cathepsin D; Olig2: oligodendrocyte transcription factor 2; O4: marker for oligodendrocyte lineage.
Ms: mouse, Rb: rabbit
IHC: immunohistochemistry, IF: immunofluorescence
